# Metagenomic Analysis of Rural Groundwater Viromes Reveals Bacteriophage Contributions to Groundwater Microbial Ecology

**DOI:** 10.1007/s00248-026-02818-y

**Published:** 2026-06-24

**Authors:** Manar-Aleslam M. Mattar, Walaa A. Eraqi, Mohamed Bakr Zaki, Akram M. Elkashlan, Khaled A. M. Abouzid, Ramy K. Aziz, Aymen S. Yassin, Ali H. A. Elbehery

**Affiliations:** 1 Department of Microbiology and Immunology, Faculty of Pharmacy, University of Sadat City, Sadat City, Egypt; 2 Department of Microbiology and Immunology, Faculty of Pharmacy, Cairo University, Cairo, Egypt; 3 Department of Biochemistry, Faculty of Pharmacy, University of Sadat City, Sadat City, Egypt; 4 Department of Biochemistry, Faculty of Pharmacy, Menoufia National University, km Cairo-Alexandria Agricultural Road, Menofia, Egypt; 5 Department of Pharmaceutical Chemistry, Faculty of Pharmacy, Ain Shams University, Cairo, Egypt; 6 Biosciences Research Laboratories, MARC for Medical Services and Scientific Research, 6th of October City, Egypt

**Keywords:** Groundwater virome, Viral ecology, Bacteriophages, Virus–host interactions, Auxiliary metabolic genes, Environmental metagenomics

## Abstract

Groundwater ecosystems host diverse microbial communities, yet the diversity and ecological roles of their associated viral genomes remain poorly characterized. Here, we investigated viral community composition, diversity, host associations, lifestyles, and auxiliary metabolic potential in groundwater from three hand pumps located in Toukh, Qalyubia, Egypt, representing distinct local surroundings and potential contamination pressures. Using complementary viral detection approaches and a quality assessment workflow, we recovered 9,534 non-redundant viral contigs spanning a wide range of viral genome quality. Taxonomic profiling revealed dominance of tailed dsDNA bacteriophages (Uroviricota/Caudoviricetes) across all pumps, with ~ 99% of contigs not assigned below the class level. Whereas the viral composition of pump 3 was distinct and its diversity was consistently higher, pumps 1 and 2 clustered together, a pattern mirrored across taxonomic scales and diversity metrics. The majority of predicted viral hosts belonged to phylum Pseudomonadota, followed by Actinomycetota, Bacillota and Bacteroidota, with levels that varied between pumps. Correlation and network analyses showed strong concordance between the relative abundance of bacteria and the abundance of viruses that potentially infect them. Lifestyle prediction indicated a descending relative abundance of viruses with lysogenic lifestyle from pumps 1 through 3. Auxiliary metabolic genes (AMGs) related mainly to nucleotide, amino acid, and cofactor metabolism were detected in all pumps, with distinct pump-specific repertoires suggesting localized viral metabolic strategies. Together, these results demonstrate that groundwater viromes are ecologically structured and highly novel, with the potential ability to modulate host metabolism, highlighting their potential role in shaping subsurface microbial communities.

**Supplementary Information:**

The online version contains supplementary material available at 10.1007/s00248-026-02818-y.

## Introduction

Groundwater is a major source of safe drinking water supply worldwide and is often preferred because it can be consumed directly and is generally regarded as more reliable than surface water [1]. At the same time, groundwater is under increasing stress because use is often poorly monitored and regulated, and withdrawals continue to rise. Agriculture is the dominant driver of depletion, with crop production accounting for ~ 83% of global groundwater depletion. Moreover, groundwater currently supports ~ 43% of global irrigation, sustaining ~ 112.9 million ha of cropland [2]. Groundwater quality is also threatened by both natural and anthropogenic pollution. For example, groundwater–rock interactions can elevate arsenic, fluoride and salinity, while human land-surface activities introduce nitrate and pesticides, untreated wastewater, industrial and healthcare effluents, and emerging contaminants, such as microplastics, some of which can persist for long periods in aquifers [3]. These pressures can reshape subsurface microbial communities and their associated viromes.

Beyond its hydrochemical characteristics, groundwater hosts diverse microbial communities that drive subsurface biogeochemical processes and influence water quality [4]. Within this hidden biosphere, viruses, particularly bacteriophages or simply phages, are expected to play important ecological roles by shaping microbial community structure and function. The continental subsurface is estimated to contain 2 to 6 × 10²⁹ prokaryotic cells [5], and viruses are a consistent companion of these microbial communities [6]. Although virus-to-prokaryote ratios vary considerably among aquifers, from near parity to more than an order of magnitude higher, viruses have been generally reported to outnumber prokaryotes in groundwater environments [7–9]. Despite several metagenomic studies investigating groundwater viral diversity, a large fraction of this diversity remains unexplored. Characterizing this understudied viral community is essential for a better understanding of groundwater quality and ecosystem functioning.

Phages shape microbial ecosystems through multiple, non-mutually exclusive mechanisms. By lysing their hosts, they redirect cellular biomass into dissolved organic matter and nutrients that become available for other microbes, thereby influencing carbon and nutrient cycling [10]. Viral predation can also regulate community structure by preferentially infecting abundant or competitively dominant taxa, a process often referred to as “Kill-the-Winner,” which can promote microbial diversity [11]. Besides lytic infection, phages can also persist through lysogeny, a strategy that may be selected when environmental conditions are unfavorable, such as during nutrient limitation or reduced host productivity [12]. In addition, phages contribute to genome evolution via horizontal gene transfer (HGT), including generalized and specialized transduction, which can disseminate traits relevant to environmental persistence and public health [13]. Finally, viral genomes may contain auxiliary metabolic genes (AMGs) that could modify host metabolism during infection, expanding the functional landscape through which viruses can affect ecosystem processes [14].

Despite these recognized roles, groundwater viromes remain far less characterized than those from marine, freshwater, soil, and host-associated ecosystems. Viral research in groundwater has long been constrained by low biomass, the lack of a universal viral marker gene, and methodological biases introduced by viral enrichment or amplification strategies. Advances in viral metagenomics and computational identification techniques are overcoming these barriers by enabling the direct recovery of viral genomes from whole-community DNA [15]. These methodological improvements have facilitated more analyses of groundwater viral communities across different aquifers. These studies indicate that groundwater viruses are highly diverse, strongly structured by local environmental conditions, and dominated by genomic novelty. For instance, a study analyzing 16 groundwater metagenomes from aquifers with varying chemical and physical properties recovered over 1,500 viral genomes, 95% of which were unclassified, and found no shared viral species across aquifers [16]. Large-scale cataloging efforts further support this view. A recent Groundwater Virome Catalogue assembled from more than 600 wells reported over 280,000 viral operational taxonomic units (vOTUs) with the vast majority lacking close matches in existing databases [6]. These findings collectively suggest that subsurface viral ecology is both globally significant and still substantially under-sampled.

In Egypt, groundwater research has largely focused on hydrochemical parameters and culture-based microbiology, leaving viral diversity and virus–host interactions poorly characterized [17]. Rural groundwater systems are often exposed to localized anthropogenic pressures, including inadequate sanitation and agricultural activity, which may influence microbial communities and their associated viruses [18, 19]. In our previous shotgun metagenomic analysis of rural hand pump groundwater from Toukh, Qalyubia, Egypt, we identified signatures of fecal contamination, detected potential bacterial pathogens, and documented antimicrobial resistance determinants [20]. However, the viral component of these groundwater metagenomes has not yet been examined.

Here, we characterized the viral communities identified in groundwater samples collected from three rural hand pumps in Toukh, Qalyubia, Egypt, representing contrasting local surroundings and potential contamination pressures. Using shotgun sequencing combined with contemporary viral genome recovery and annotation workflows, we aimed to (i) profile viral diversity and community composition across sampling sites, (ii) identify dominant viral taxa and assess the distribution of predicted lytic and lysogenic lifestyles, (iii) predict putative prokaryotic hosts to explore virus–host associations and their agreement with host community patterns, and (iv) identify candidate AMGs to evaluate the potential for viral modulation of microbial metabolism. To our knowledge, no metagenome-based characterization of groundwater viromes from Egypt is publicly availble. This study will potentially contribute to the growing global understanding of subsurface viral ecology.

## Materials and Methods

### Sample Collection and Sequencing

Groundwater samples were previously collected from three groundwater hand pumps located in Toukh, Qalyubia, Egypt [20]. The sampling sites represented distinct local surroundings: pump 1 (30°20′27.14″ N, 31°11′37.69″ E) was situated near cultivated farmland and a cemetery, pump 2 (30°20′42.69″ N, 31°11′46.35″ E) adjacent to an agricultural field and residential area that uses sewage-holding tanks instead of a municipal sewer network, and pump 3 (30°20′56.40″ N, 31°12′15.35″ E) close to a residential area supplied with a regular sewer system. Three independent replicates typically collected on three successive days were obtained from each pump, yielding a total of nine groundwater samples. Each groundwater sample consisted of approximately 10 L collected in sterile containers, with sampling conducted in the early morning (approximately 6:00–7:00 AM) during the dry season (spring to early summer, March–July 2023). Water samples were subjected to 0.2 μm membrane filtration, after which total DNA was extracted using DNeasy PowerSoil Kit (Qiagen, Valencia, USA) following the manufacturer’s instructions [20]. High-throughput paired-end sequencing on Illumina NovaSeq 6000 with a read length of 150 bp was done by Novogene (Beijing, China). No virus-like particle (VLP) concentration step was performed prior to DNA extraction; instead, viral sequences were identified in silico from total metagenomic data (see below).

### Sequence Preprocessing and Assembly

Fastp (v0.23.2) was used with the default parameters for quality control and adapter removal of raw sequence reads [21]. To improve assembly quality and contig recovery, we assembled reads from the three replicates of each pump into contigs using MEGAHIT v1.2.9 [22] with default parameters. Sequence reads were separately assembled for each pump rather than independently per replicate; therefore, replicate independence applies to abundance estimation but not to contig generation.

### Viral Contig Identification and Quality Assessment

Using BBTools reformat.sh v24.04.3 [23], we removed assembled contigs shorter than 1,000 bp. Viral sequence identification was independently performed with geNomad v1.8.0 [24] (end-to-end mode, conservative option, score calibration enabled, and composition set to metagenome) and VirSorter2 v2.2.4 [25] with default parameters. To assess the completeness and contamination of predicted viral sequences, contigs from each prediction tool were analyzed separately by CheckV v1.0.3 [26] with default parameters, together with its database v1.5. To avoid false positive predictions, we flagged and excluded contigs containing only host genes and no viral genes (based on CheckV contamination reports). The remaining viral and proviral sequences reported by CheckV were retained for downstream analyses. The filtered outputs from both tools (geNomad and VirSorter2) were then concatenated, and clustered by CD-HIT-EST v4.8.1 [27] at 95% sequence identity threshold, word length of 10 and a coverage of 85% on the shorter sequence yielding representative vOTUs for downstream analyses, according to previously published standards [28]. vClust v1.3.1 [29] was used to validate clustering results, with a minimum average nucleotide identity (ANI) of 95% and query coverage (aligned fraction, AF) of 85%, producing identical representative vOTUs. To examine the overlap of vOTUs across pumps, we concatenated vOTUs from all pumps and clustered them using vClust v1.3.1 [29] at 95% ANI and 85% AF.

### Abundance Profiling

Relative abundance values were estimated as reads per kilobase per million reads (RPKM) by mapping reads from each replicate separately to viral contigs from their corresponding pump using BBMap v24.04.3 with default parameters [30].

$$\:RPKM=\:\frac{number\:of\:mapped\:reads}{contig\:length\:\left[kb\right]\times\:total\:number\:of\:reads\:\left[million\right]}$$

### Taxonomic Classification, Lifestyle and Host Prediction

Viral sequences were taxonomically classified by the Contig Annotation Tool (CAT) v6.0.1, with default parameters [31], against a custom viral protein database from both GenBank and RefSeq (downloaded on January 30, 2025, and including 55,559,602 viral proteins). All filtered non-redundant viral contigs across all CheckV quality tiers were used for this classification. Additionally, PhaTYP [32] was used with default parameters to predict virus lifestyle (lytic or lysogenic) for viral contigs classified by CheckV as medium-quality, high-quality, or complete viral genomes (172 in total). To test whether phage lifestyle distributions differed among pumps (pumps 1–3), we performed a chi-square test of independence in R, followed by Holm correction test [33]. A genome-based proteomic tree was constructed by VIPTree v4.0 [34] for medium-quality, high-quality, and complete genomes (quality determined by CheckV), in addition to reference viral genomes from NCBI RefSeq collected by VIPTree based on a minimal similarity score of 0.1. The tree was visualized using iTOL v7.2.2 [35]. Moreover, to predict the prokaryotic hosts for the identified viral contigs, we used iPHoP v1.3.3 [36] for all filtered non-redundant viral contigs for each pump. The default confidence score cutoff (≥ 90) was applied to retain high-confidence host predictions. All heatmaps show normalized abundances on a log_10_(RPKM) scale.

### Correlation and Network Analysis of Bacterial and Viral Host Abundances

Prokaryotic host genera for viral contigs were predicted by iPHoP (August 2023 database release) [36]. For each pump, the RPKM of individual viral contigs was matched to their corresponding host genus and summed to yield genus-level RPKM profiles representing the abundance of viruses predicted to potentially infect each host genus. For each pump, the correlation analysis was restricted to the intersection of the 100 most abundant genera in the Bracken-derived bacterial profiles [20] and the iPHoP-based predicted viral-host profiles. This approach avoids stochasticity associated with rare community members. After centered log-ratio transformation of Bracken (%) and iPHoP (RPKM) values, data were visualized as scatter plots for each pump with ggplot2 v3.5.1 [37], and Pearson and Spearman correlation coefficients were calculated with the base R *stats* package [33]. To further examine virus–host relationships, pump-specific bipartite networks were generated and visualized in Gephi Lite v1.0.1 [38].

### Auxiliary Metabolic Gene (AMG) Detection

AMG prediction was carried out by the DRAM-v pipeline v1.5.0 with default parameters [39]. All filtered non-redundant viral contigs across all CheckV quality tiers were first prepared with VirSorter2 (using --prep-for-dramv option) [25], then annotated with DRAM-v annotate, followed by functional summarization with DRAM-v distill. We retained metabolic AMGs identified by DRAM-v, including known and experimentally supported AMGs, while excluding candidates with strong viral structural, host contamination, or mobile element signals. To increase confidence in AMG identification, we applied DRAM-v auxiliary score filtering, which evaluates the genomic context of each gene based on the presence of viral hallmark or viral-like genes in flanking regions. Lower auxiliary scores indicate stronger viral support (e.g., flanking viral hallmark or viral-like genes), whereas higher scores indicate weaker or ambiguous viral context, including potential host-derived regions [25]. Only AMGs with auxiliary scores < 4 were retained, ensuring that selected genes were supported by a genomic context consistent with viral origin. To refine auxiliary metabolic gene (AMG) identification, we applied an additional filtering step to exclude genes associated with core viral functions, including nucleotide metabolism, DNA replication, and DNA modification. This curation follows current recommendations for AMG annotation [40] and ensures that retained genes are more likely to represent host metabolic modulation.

To further study the viral dimethyl sulfoxide reductase subunit A (*dmsA*), we examined its genomic context using Pharokka [41] annotations and visualized it using LoVis4u [42]. Reference DmsA protein sequences were retrieved from UniProtKB [43], with the keyword “dmsA,” and filtered to remove non-specific and partial entries. Redundant sequences were reduced by CD-HIT [27] at 90% identity, followed by further dataset size reduction with Treemmer v0.3 [44], set to a relative tree length (RTL) of 0.95. The final number of DmsA references was 197. MAFFT v7.525 [45] was used for multiple sequence alignment. A maximum-likelihood phylogenetic tree of DmsA sequences was constructed by IQ-TREE v3.0.1 [46], with automatic model selection and 1,000 ultrafast bootstrap replicates, including four nitrate reductase (NarG) sequences as an outgroup. Conserved domains and catalytic features of the viral DmsA were assessed using InterPro [47].

### Viral Genome Annotation

Pharokka v1.7.2 [41] was used for genome annotation of all filtered non-redundant viral contigs across all CheckV quality tiers. The program was set to the ‘meta mode’, with Prodigal-GV for gene prediction and the --meta_hmm option to use both MMseqs2 [48] and PyHMMER [49] for gene annotations.

DRAM-v and Pharokka annotations were used for distinct purposes (AMG identification and general genome annotation, respectively) and were not integrated at the gene level.

### Diversity Analysis

Alpha diversity was assessed at the species level with the Shannon index and richness calculated with the phyloseq package v1.48.0 [50], while evenness was estimated with the vegan package v2.7.0 [51]. The statistical significance of alpha diversity metrics was tested by Welch’s ANOVA, followed by Games-Howell posthoc test. Beta diversity was calculated with the Bray-Curtis dissimilarity metric using phyloseq v1.48.0. Species-level RPKM values from CAT analysis for each replicate separately served as inputs for these calculations to test the inter-pump differences. Thus, within-pump variability was represented by the three independent replicates from each pump. Visualizations were produced using ggplot2 v3.5.1 [37].

### vConTACT3 Analysis

Medium-quality, high-quality, and complete viral genomes were clustered using vConTACT3 v3.1.3 [52] and compared against the RefSeq viral genome database (version 230) using default parameters. vConTACT3 inferred clusters based on shared protein content and assigned taxonomic affiliations.

## Results

### Assembly of High-quality Viral Contigs From Metagenomic Reads

The main goal of this study was to estimate the composition and diversity of viral sequences in the metagenomes of groundwater from three hand pumps with different exposure to pollution. To this end, we used two complementary viral-identification tools, namely geNomad and VirSorter2 and recovered multiple viral contigs, which we checked for quality using CheckV. Viral contig quality ranged from short fragments to complete viral genomes. Pump 1 yielded 4 complete, 26 high-quality, and 41 medium-quality contigs; pump 2 yielded 3 complete, 15 high-quality, and 28 medium-quality contigs; and pump 3 yielded 6 complete, 6 high-quality, and 43 medium-quality contigs. Mean contig length decreased with quality tier: complete, high-quality, medium-quality, and low-quality contigs averaged 58,164 bp, 38,892 bp, 33,513 bp, and 2,897 bp, respectively (Supplementary Table 1).

### Distinct Taxonomic Composition of the Viromes

Most of the recovered viral contigs were assigned to the superkingdom Viruses, but a considerable fraction (31.8–49.7%) remained unclassified, probably because of sequence novelty or limited representation of environmental viral genome sequences in the current databases (Supplementary Figure S2).

Across all pumps, assigning taxonomic classification to recovered viral contigs was more successful at higher ranks (phylum, class). At the phylum level, 1,867 (91.0%), 1,427 (93.6%), and 4,576 (92.9%) contigs could be classified in pump 1, pump 2, and pump 3, respectively with only a small fraction remaining unclassified (82, 52, and 259 contigs, respectively). In contrast, the resolution of taxonomic classification dropped at lower ranks. For example, at the order level, only 71 (pump 1), 52 (pump 2), and 291 (pump 3) contigs could be assigned, with the majority of contigs remaining unclassified at this rank (Supplementary Table 2).

At the phylum level, hierarchical clustering grouped pump 1 and pump 2, whereas pump 3 formed a distinct cluster, indicating a compositional difference between pump 3 and the other two pumps (Supplementary Figure S3A). Across all pumps, the virome was dominated by Uroviricota (head-tailed dsDNA phages) – RPKM values: pump 1, 2,241.4; pump 2, 1,795; pump 3, 4,851. Two additional phyla were consistently detected at lower and pump-specific abundances: Hofneiviricota (ssDNA viruses), which was detected in pump 1 (RPKM: 26.7) and pump 2 (RPKM: 54.2), and Nucleocytoviricota (large eukaryotic DNA viruses), which was relatively enriched in pump 3 (147.7 RPKM) but less abundant in pump 1 (45.1 RPKM) and minimal in pump 2 (19.4 RPKM) (Figure S3A-C).

At the class level, clustering was consistent with the phylum-level pattern, grouping pumps 1 and 2 together, while separating pump 3 (Supplementary Figure S3B). Caudoviricetes dominated all pumps (RPKM values: pump 1, 2,241.4; pump 2, 1,795; pump 3, 4,851), with Megaviricetes (giant viruses) and Faserviricetes (ssDNA bacteriophages) present at lower relative abundances. Pump 1 exhibited higher Megaviricetes (44.1 RPKM) followed by Faserviricetes (26.7 RPKM). Similarly, pump 3 showed a relatively high Megaviricetes abundance with RPKM of 145.8 and a minimal Faserviricetes presence (2.3 RPKM). On the other hand, pump 2 showed the opposite pattern (Faserviricetes 54.2 RPKM, Megaviricetes 19.4 RPKM).

At lower taxonomic levels, most Caudoviricetes-assigned viral contigs (99.1% in pump 1, 99.7% in pump 2 and 99.9% in pump 3) could not be classified, indicating potential taxonomic novelty. Consistent with the phylum- and class-level taxonomic profiles, species-level clustering grouped pumps 1 and 2 together, with pump 3 forming a distinct cluster (Supplementary Figure S3C). Pump 1 was characterized by a distinct profile, enriched in phages linked to soil, sediment, anaerobic, or host-associated bacteria, such as several *Geobacillus*/*Burkholderia* phages, Verrucomicrobia phage, and methanogenic archaeal viruses (Supplementary Figure S4A). Distinctly, the viral distribution in pump 2 was of higher abundance of viruses similar to *Shigella* phage SfIV, *Thermus* phage phi OH2, and several *Burkholderia* and other host-associated phages (Supplementary Figure S4B). Pump 3 was characterized by elevated abundances of sequences with high similarity to phages associated with oligotrophic or environmental hosts, including *Azospirillum* phage Cd, *Pelagibacter* phage HTVC008M, *Thermoanaerobacterium* phage THSA-485 A, *Puniceispirillum* phage HMO-2011, and Cyanophage PSS2 (Supplementary Figure S4C). Together, these results emphasize that pumps 1 and 2 are more phylogenetically related to one another than to pump 3, which has been consistently distinct, regardless of the compared taxonomic features. The divergence of pump 3 likely reflects differences in host community structure and/or environmental inputs relative to the more similar conditions shared by pumps 1 and 2 [20].

### Differential Viral Diversity Across the Three Pumps

**Fig. 1:**
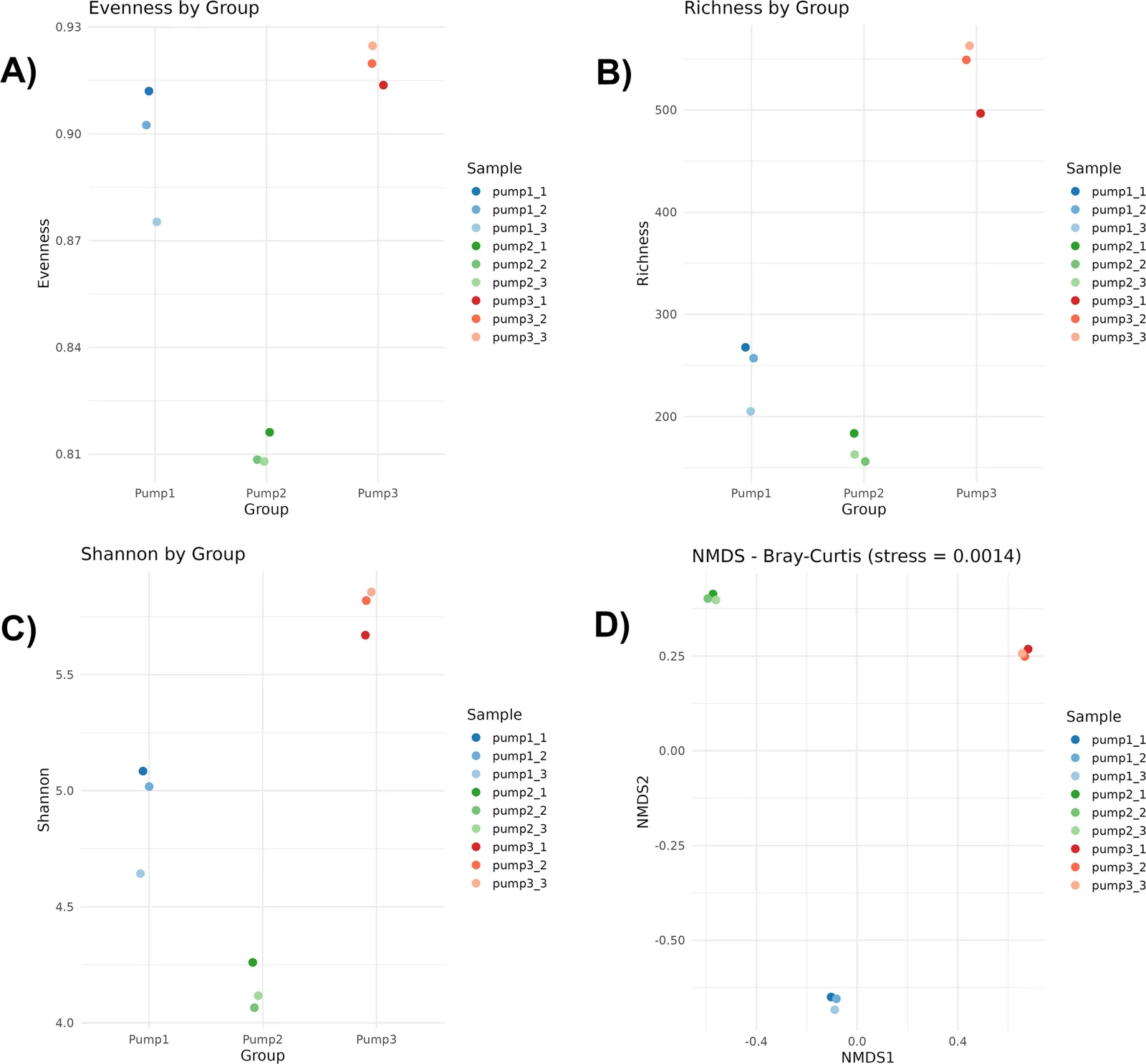
Alpha and beta diversity. Dot plots of species-level alpha diversity metrics by sample: (**A**) evenness, (**B**) richness, and (**C**) Shannon index. Non-metric Multidimensional Scaling (NMDS) plot illustrating beta diversity assessed using Bray-Curtis dissimilarity distance for pumps 1, 2, and 3 (**D**)

Among the three pumps, pump 3 had the highest values of alpha diversity at the species level, regardless of which metric was used (Shannon index, richness, and evenness). *Post hoc* comparisons showed that the Shannon index of pump 3 was significantly higher than that of pump 1 and pump 2 (adjusted p-values (p_adj_) of 0.026 and 0.0000847, respectively). Richness was likewise higher in pump 3 than in pump 1 and pump 2 (p_adj_ = 0.001 and 0.002, respectively), and pump 1 also had a richer viral community than pump 2 (p_adj_ = 0.034). Moreover, both pump 3 and pump 1 had significantly higher evenness than pump 2 (p_adj_ = 0.0000496 and 0.001, respectively, Fig. 1A-C).

Beta diversity was estimated by the Bray-Curtis measure of dissimilarity and visualized through an NMDS plot. Replicates from each pump clustered tightly together and remained distinct from other pumps, confirming that inter-pump variability across the viral communities was higher than intra-pump variability (Fig. 1D). NMDS ordination (two dimensions, k = 2) yielded a stress value of 0.0014, indicating an excellent fit between the 2D representation and the original data. Shared vOTUs across pumps were minimal, with only 0.66% of the total vOTUs detected in more than one pump, confirming the beta diversity results and suggesting that the virome in each pump is site-specific.

### Novelty Among Viral Genomes

High- and medium-quality viral genomes (172 contigs) were clustered together, in reference to RefSeq viral genomes, with vConTACT3. vConTACT3 uses gene content clustering and machine learning, with the ability to reach high accuracy clusters, largely corresponding to ICTV taxonomy particularly across five ranks: realm, order, family, subfamily and genus. At the realm rank, 70.9% of the genomes (122 out of 172) were classified as Duplodnaviria, while only four genomes (2.3%) were classified as Monodnaviria, and the remaining genomes (46 genomes, 26.7%) were regarded as singletons, i.e., could not be classified for this or lower ranks (Supplementary Figure S5). At the order level, only four genomes (2.3%) could be assigned to Tubulavirales, the remaining genomes (122 out of 172, 70.9%) were assigned to 50 different novel orders. At the family level, five genomes (2.9%) were assigned to Peduoviridae, four (2.3%) to Inoviridae, two (1.2%) to Autographiviridae, and one (0.6%) to Saffermanviridae. The remaining 114 genomes (66.3%) were assigned to 92 novel families. At the subfamily level, all genomes (126 genomes, 73.3%) were assigned to novel subfamilies. At the genus level, only two genomes (1.2%) were assigned to known genera, namely *Mycoabscvirus* and *Siphunculivirus*, while the remaining 124 genomes (72.1%) were assigned to 122 novel genera. Since singleton genomes have no significant similarity to other genomes, they represent potentially novel genomes. Adding up singleton genomes to novel genomes, at each rank, yielded 168 genomes (97.7%) at the order level, 160 genomes (93.0%) at the family level, 172 genomes at the subfamily level (100%), and 170 genomes (98.8%) at the genus level.

### Viral Host Predictions Across Pumps

**Fig. 2:**
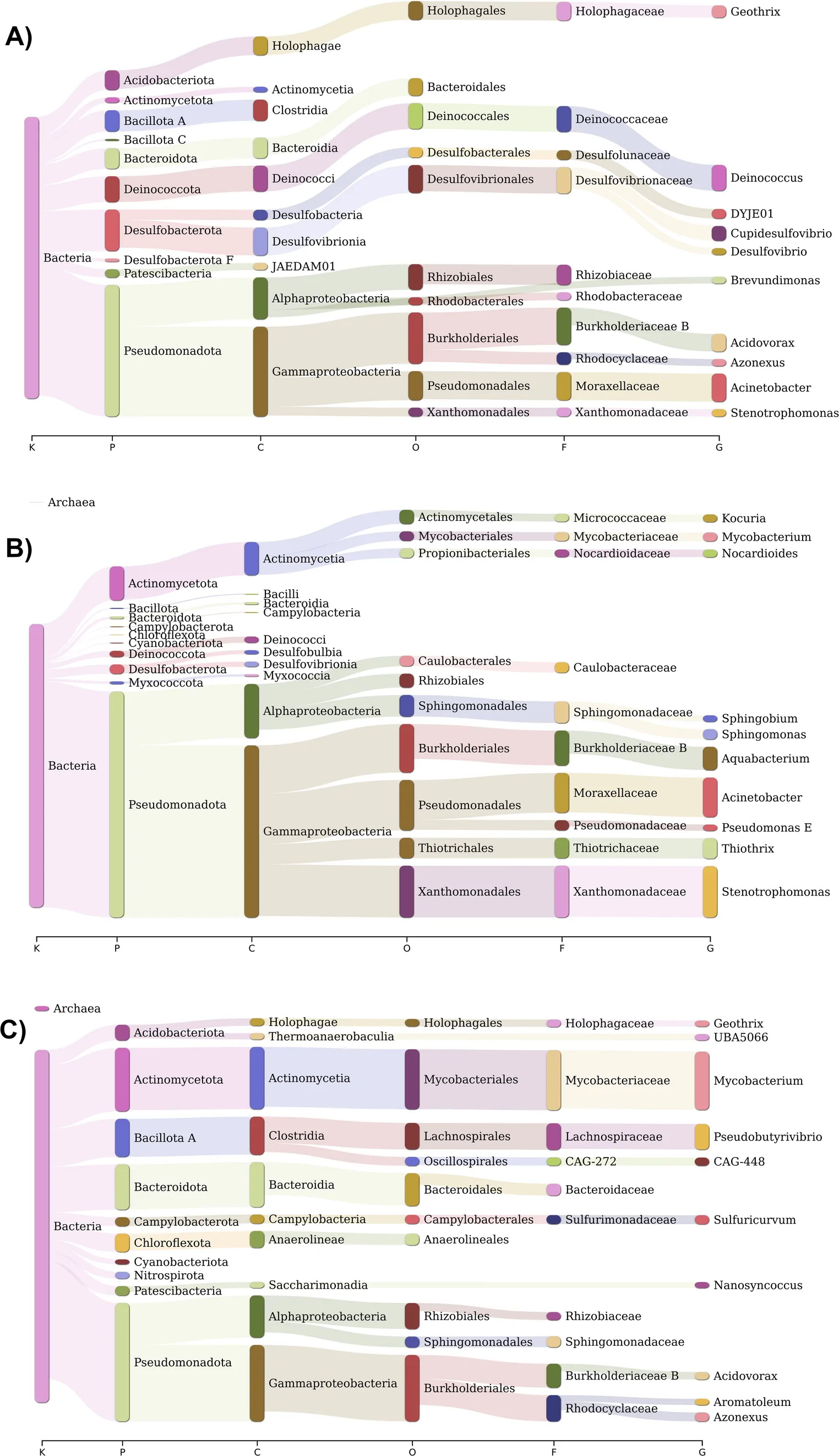
Sankey diagram (generated by Pavian) showing the taxonomic classification of viral host predictions across pump 1 (**A**), pump 2 (**B**), and pump 3 (**C**)

Across pumps, the most frequently predicted viral hosts were members of the phylum Pseudomonadota (formerly Proteobacteria), with the highest abundance in pump 2 (929.51 RPKM), followed by pump 1 (448.77 RPKM) and pump 3 (151.36 RPKM, Fig. 2A-C). In pump 1, Pseudomonadota was followed in frequency by Desulfobacterota (141.09 RPKM) and Deinococcota (87.84 RPKM). In pump 2, the second most abundant phylum was Actinomycetota (139.49 RPKM), while Desulfobacterota and Deinococcota were comparatively lower (38.33 and 25.01 RPKM, respectively). The broadest host diversity was observed in pump 3: Pseudomonadota members remained the most frequently predicted hosts, followed by Actinomycetota (81.03 RPKM), Bacteroidota (57.98 RPKM), and Bacillota_A (48.57 RPKM), with lower frequency of Campylobacterota (11.38 RPKM; sulfur-oxidizing lineages). Patescibacteriota (Candidate Phyla Radiation, CPR) were detected as predicted hosts in pumps 1 and 3 (27.88 and 11.95 RPKM, respectively), consistent with virus–host associations in low-energy, carbon-limited groundwater niches.

At the class level, Gammaproteobacteria was the most frequently predicted host class in pumps 1 and 2 (306.52 and 707.82 RPKM, respectively), followed by Alphaproteobacteria (142.26 and 221.69 RPKM, respectively). In pump 3, Gammaproteobacteria were similarly more frequently predicted (97.64 RPKM), with Actinomycetia as the second most abundant class (79.52 RPKM).

At the family level, more prominent differences could be observed between predicted viral hosts. For example, the host lineages possibly implicated in sulfur cycle differed by pump. Specifically, in pump 1, a potential sulfate-reducer signal was represented by the predicted host families *Desulfovibrionaceae* (89.7 RPKM) and *Desulfolunaceae* (32.2 RPKM), suggesting a viral community that is associated with hosts with potential sulfate reduction capacity. Family *Desulfovibrionaceae* was also predicted as a potential host family for viral genomes from groundwater samples in pump 2, but at a lower abundance (19.0 RPKM), all of which belonged to genus *Humidesulfovibrio*, formerly known as *Desulfovibrio*. In addition, pump 2 was characterized by predicted sulfur-oxidizing hosts, e.g., family *Thiothrichaceae* (83.8 RPKM), members of which commonly perform nitrate-coupled sulfur oxidation under microoxic conditions. Similarly, viral sequences from pump 3 were predicted to infect *Sulfurimonadaceae* (*Sulfuricurvum*-like oxidizers, with relative abundance of 11.4 RPKM), indicating potential sulfur oxidation coupled to nitrate reduction.

Moreover, predicted viral hosts likely supporting the nitrogen cycle were present in all pumps e.g., *Burkholderiaceae* and *Rhizobiaceae*, whose members are widely known for nitrate reduction/denitrification potential, both of which were predicted in all three pumps, but with higher abundances in pumps 1 and 2 compared to pump 3 (*Burkholderiaceae*: 126.8, 143.4, and 30.5 RPKM; *Rhizobiaceae*: 68.5, 26.9, and 10.1 RPKM at pumps 1, 2, and 3, respectively), suggesting a viral community associated with microbial hosts with a potential for biological nitrogen fixation. In addition, viral sequences from pumps 2 and 3 were predicted to have *Sphingomonadaceae* hosts with much less abundance at pump 1 (1.98, 88.7, and 13.9 RPKM at pumps 1, 2, and 3, respectively). Members of this family are known to often denitrify under low oxygen, while degrading aromatic compounds.

### Correlation and Network Analysis of Bacterial and Predicted Viral Host Abundances

**Fig. 3:**
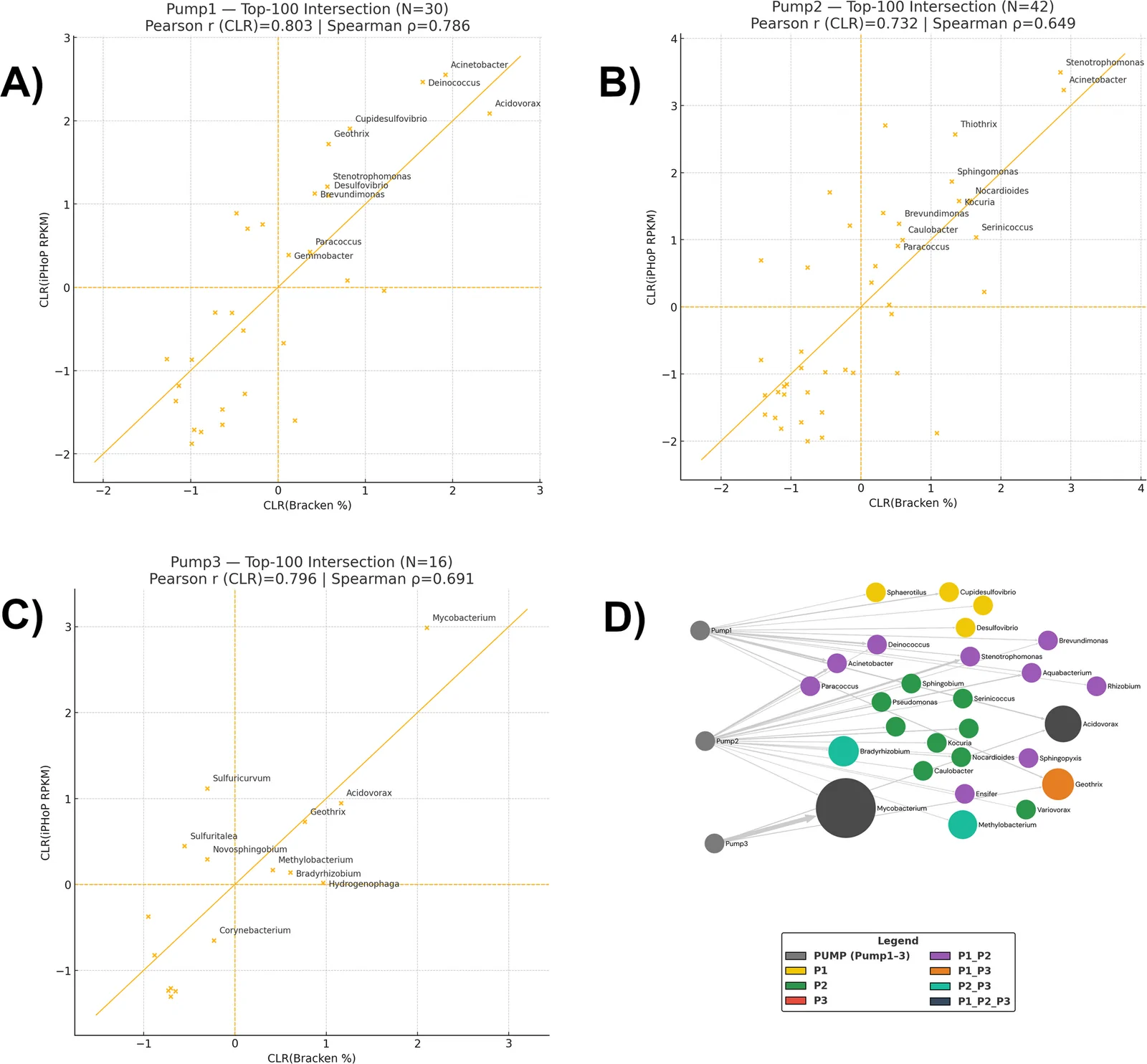
CLR-transformed scatter plots showing the relationship between bacterial genus abundances (Bracken, %) and the abundance of viral contigs predicted to have the same bacterial host genera (iPHoP, RPKM) for (**A**) Pump 1, (**B**) Pump 2 and (**C**) Pump 3. Pearson and Spearman correlation coefficients are reported for each pump. (**D**) Bipartite virus–host network illustrating connections between groundwater pumps and bacterial genera, with node size proportional to Bracken-derived bacterial abundance and edge thickness proportional to the abundance of viral contigs predicted to have the same bacterial host (total RPKM) for each pump–genus pair. Only a subset of genera is labeled in panels A–C for readability, but all plotted genera were included in the correlation analysis regardless of labeling

To test whether the actual groundwater microbial community structure [20] is correlated with the predicted hosts for the identified viral contigs in this study, we conducted a correlation analysis between genus-level microbial relative abundances (from our previous study [20]) and RPKM values of the viral contigs predicted to have the same host genera. For each pump, the analysis was restricted to the intersection of the top 100 most abundant genera in both datasets, yielding 30, 42 and 16 shared genera for pump 1, pump 2, pump 3, respectively. After centered-log ratio (CLR) transformation, strong positive correlations were observed between the abundances of bacterial genera and viruses predicted to be associated with these genera in all pumps (Fig. 3A-C). Pearson correlation coefficients ranged from 0.73 to 0.80 (pump 1: *r* = 0.80, 95% CI 0.65–0.90; pump 2: *r* = 0.73, 95% CI 0.52–0.87; pump 3: *r* = 0.80, 95% CI 0.45–0.94), and Spearman’s ρ showed a similar pattern (0.65–0.79), indicating a strong positive correlation between identified genera that are abundant in the bacterial community and relative abundance of viruses that potentially infect them. In other terms, abundance patterns of viruses identified in each community are consistent with bacterial host abundances, suggesting potential virus–host associations, although these correlations may also reflect shared environmental drivers rather than direct predator–prey dynamics.

A bipartite virus–host network further illustrated how these relationships are distributed across pumps (Fig. 3D). Pump-specific nodes (bacterial hosts) highlighted genera whose viral signal (viral contigs predicted to have the same bacterial host) was restricted to a single pump. Alternatively, other nodes had viral signals shared between two or all three pumps. Within this core set, *Mycobacterium* and *Acidovorax* formed prominent hubs, characterized by large node sizes (high bacterial abundance) and thick edges (strong viral signal), and present in all pumps. Overall, the combined correlation and network analyses show that predicted viral host profiles largely mirror the underlying bacterial community composition, while also highlight a smaller group of key host genera that repeatedly appear and associate with viruses that potentially infect them across the three pumps. These patterns suggest possible repeated virus–host associations, but they do not directly demonstrate stable interactions.

### Virus Lifestyles Across Pumps

**Fig. 4:**
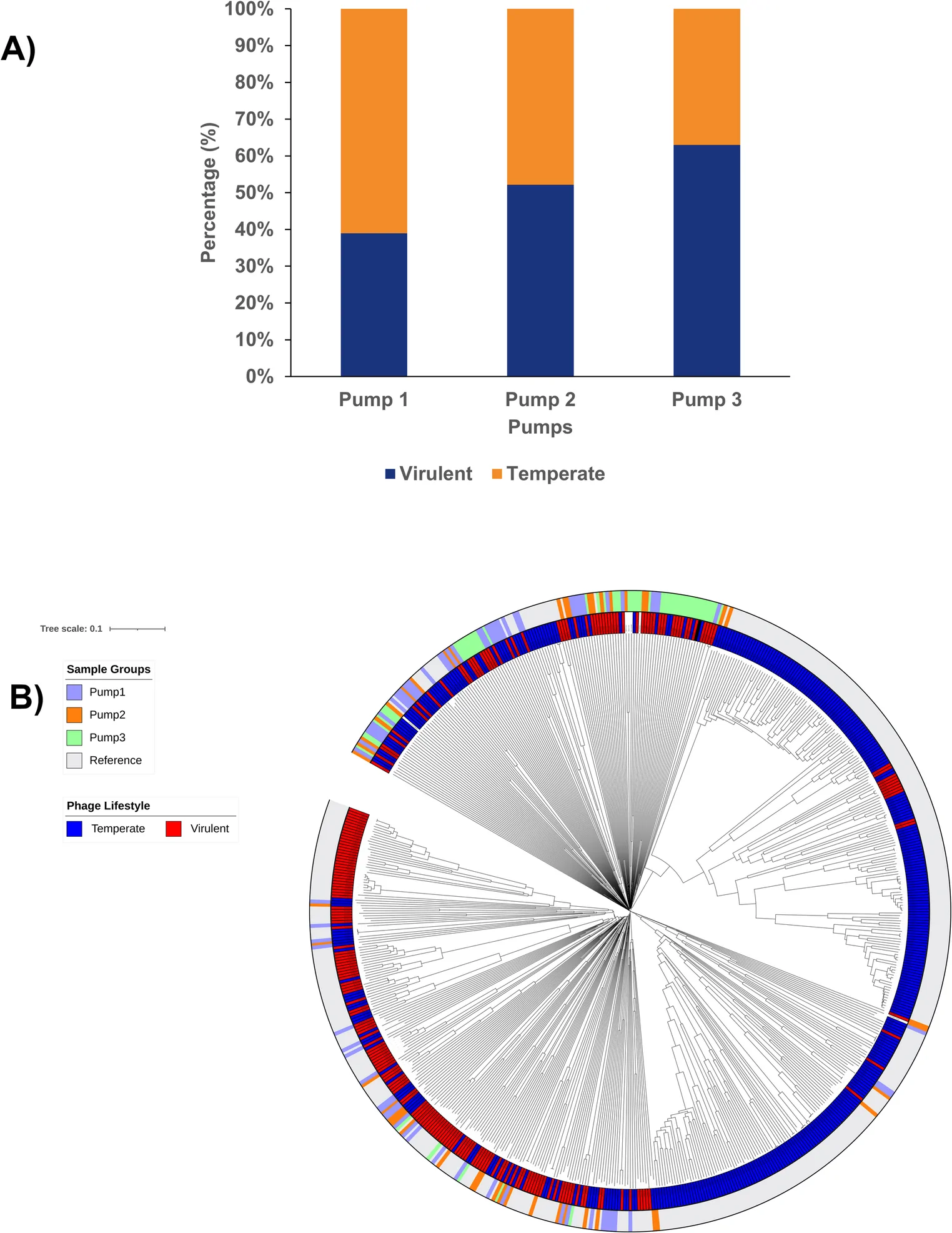
Predicted lifestyle of identified viral genomes across the three pumps. (**A**) Stacked bar chart showing predicted viral lifestyles across pump 1, pump 2 and pump 3. A chi-square test of independence indicated that lifestyle composition significantly differed among pumps (χ² = 10.46, *p* = 0.005). *Post hoc* comparisons with Holm correction showed a significant difference between pump 1 and pump 3 (adjusted *p* = 0.008). (**B**) Phylogenomic tree of groundwater viral genomes, annotated with predicted phage lifestyle (inner ring). A temperate virus is one that switches between lytic and lysogenic cycles, while a virulent virus is one predicted to be strictly lytic

The lifestyles of groundwater viruses (lytic or lysogenic) were predicted by PhaTYP [32], which uses deep learning to predict viral lifestyle based on predicted protein composition and interactions from phage contigs. Overall, all high- and medium-quality viral contigs were classifiable: viral contigs associated with a lysogenic lifestyle were more abundant in pump 1 (61%) and slightly more abundant in pump 2 (53%) than in pump 3 (37%). This pattern may suggest lower lytic pressure in groundwater from these pumps and may be associated with the dominance of fewer taxa and lower microbial diversity detected in groundwater samples from these two pumps [20]. Conversely, 63% of viral contigs identified in pump 3 were predicted to belong to strictly lytic phages (63%, Fig. 4A), a proportion that significantly differed from pump 1 (Chi-Square followed by Holm adjustment, *p* = 0.008). This finding may suggest a top-down viral control that may limit dominance of some microbial taxa over others and promote higher microbial community evenness, as observed in groundwater samples from pump 3. A phylogenomic tree of these viral genomes did not have segregation or separate clustering of lytic versus temperate phages (Fig. 4B).

### Auxiliary Metabolic Genes (AMGs)

**Fig. 5:**
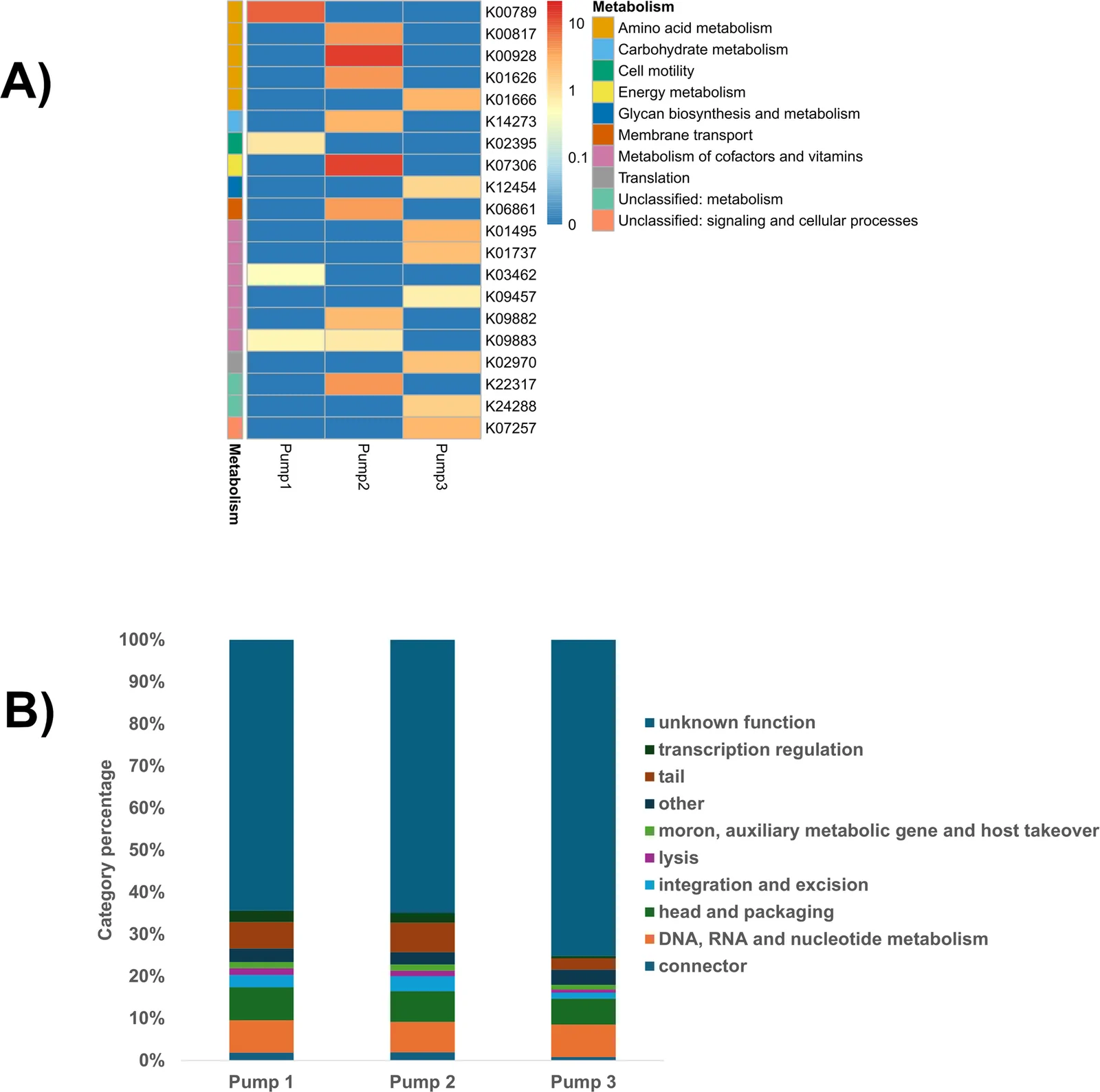
Functional annotation of identified viral genomes. (**A**) Heatmap showing the relative abundance (RPKM) of auxiliary metabolic genes detected in groundwater samples from the studied pumps (pump 1, pump 2, and pump 3). Each row represents a KEGG orthology (KO) identifier. Row annotations indicate metabolic categories based on KEGG BRITE level 2 classification. RPKM values were log10-transformed after adding a pseudo-count of 0.01 for visualization; legend labels correspond to the original RPKM values. (**B**) Distribution of the functional categories assigned to viral gene products across the three pumps

AMGs were identified in viral communities from groundwater from all three pumps, although their abundance and functional composition varied among sites (Fig. 5A). Across all pumps, AMGs were dominated by genes involved in amino acid metabolism, followed by cofactor and vitamin metabolism, energy metabolism, and other functional categories.

Amino acid metabolism–related AMGs displayed strong pump-specific distributions. For example, S-adenosylmethionine synthetase (K00789) was detected exclusively in pump 1, whereas groundwater from pump 2 contained several amino acid metabolic enzymes, including aspartate kinase (K00928), histidinol-phosphate aminotransferase (K00817), and 3-deoxy-7-phosphoheptulonate synthase (K01626). Groundwater from pump 3 contained a distinct amino acid metabolism AMG, 4-hydroxy-2-oxovalerate aldolase (K01666), which was absent from the other pumps.

Functional differences among pumps were also evident in other metabolic categories. Energy metabolism–related AMGs were detected only in groundwater from pump 2, represented by anaerobic dimethyl sulfoxide reductase subunit A (K07306, *dmsA*). Genomic context analysis showed that the DmsA-encoding gene is embedded within a clear viral genomic context containing hallmark phage genes (e.g., head maturation protease, endolysin, recombinase), confirming its viral origin. The *dmsA* gene is also co-localized with adjacent ferredoxin-encoding genes, consistent with a redox-related functional module (Supplementary Fig. S6A). Phylogenetic analysis demonstrated that the viral DmsA clusters well with other reference DmsA proteins and is closely related to DmsA from *Candidatus Venteria ishoeyi*, while remaining clearly distinct from the NarG outgroup (Supplementary Fig. S6B). Domain analysis further confirmed the presence of the domains and residues typical of dimethylsulfoxide reductase proteins (Supplementary Fig. S6C), supporting the functional annotation of this gene.

In contrast, pump 3 was characterized by a broader representation of cofactor and vitamin metabolism, including enzymes involved in folate and pterin biosynthesis (K01495, K01737, K09457). Translation-related and cellular process–associated AMGs were detected exclusively in groundwater from pump 3, whereas in groundwater from pump 1 a cell motility–associated peptidoglycan hydrolase (K02395) was uniquely detected.

Overall, while groundwater from the three pumps shared a small set of high-abundance AMGs, particularly those related to amino acid metabolism, each pump exhibited a distinct AMG profile characterized by unique functional categories and gene repertoires, indicating site-specific viral metabolic strategies in the groundwater environment.

### Functional Annotation of Viral Contigs

After functional annotation of viral contigs using Pharokka, the majority of predicted genes across all groundwater samples were of unknown function (64.4%, 64.9%, and 75.2% of annotations in pump 1, pump 2, and pump 3, respectively, Fig. 5B). Meanwhile, among the functionally annotated genes, categories associated with phage structure and replication were consistently present, including head and packaging (6.2–7.8%), tail (2.7–7.0%), and DNA, RNA, and nucleotide metabolism (7.2–7.7%). Genes involved in integration and excision were slightly more abundant in pump 2 (3.6%) and pump 1 (2.9%) compared to pump 3 (1.4%), consistent with a potentially higher contribution of temperate phages in groundwater from these two pumps. Auxiliary and host-interaction–related functions, including moron, auxiliary metabolic gene, and host takeover categories, represented a smaller but stable fraction across pumps (1.1–1.4%). Collectively, these results indicate a viral community typically enriched in genes with uncharacterized functional potential, with conserved representation of core phage structural and replication modules across groundwater pumps.

## Discussion

Despite the increasingly recognized role of viruses in shaping groundwater microbial communities, they remain understudied [53]. In this study, we provide a genome-resolved characterization of groundwater viral communities across samples from three spatially distinct rural groundwater hand pumps with different surrounding environments, revealing strong site-specific structuring in viral diversity, taxonomy, host associations, lifestyles, and functional potential. By integrating these different aspects of viral genome analysis, we show that viral communities differed across the three sampled groundwater pumps, reflecting localized ecological conditions and host community configurations.

Across all pumps, viral genomes predominantly belonged to tailed double-stranded DNA phages (Caudoviricetes), consistent with previous observations from groundwater environments [16, 53]. This dominance is not unique to groundwater; Caudoviricetes are widely recognized as the most abundant group of DNA viruses across nearly all studied ecosystems [54–56]. Likewise, the limited taxonomic resolution below the class level is a feature generally observed in most studied viromes [57], reflecting a clear gap in current viral reference databases [58]. In addition, vConTACT3 clustering revealed that the majority of high- and medium-quality genomes represent previously uncharacterized viral orders, families and genera.

While this lack of clustering with RefSeq genomes suggests potential novelty, this finding is largely database dependent. Further comparisons with expansive environmental datasets, such as IMG/VR [59] or the Groundwater Virome Catalog [6], would likely provide a more accurate assessment of viral novelty. Overall, this potential novelty observed in the studied groundwater samples aligns with the results obtained from a large-scale groundwater virome study, where more than 99% of recovered vOTUs were found to be novel upon comparison to the Integrated Microbial Genomes/Virus (IMG/VR) database and 94% could not be taxonomically annotated below the Class level [6]. Another study showed that vConTACT2 was unable to classify 95% of the recovered high-quality groundwater vOTUs, which comprised most of the viral abundance (55.1–99.6%) [16].

Groundwater viral communities from pump 3 had the highest alpha diversity, with significantly different Shannon index, richness, and evenness compared to pumps 1 and 2, indicating a virome that is both more species-rich and more uniformly distributed across taxa. This viral diversity agrees with the microbial diversity previously shown in groundwater from these pumps [20], consistent with the agreement between microbial and viral diversities shown in other environments as well [60, 61]. The difference in groundwater viral diversities among the three pumps likely mirrors the differences in environmental conditions, surroundings and potential contamination. Notably, the higher viral diversity observed in pump 3 coincides with its location near a residential area served by a conventional sewer system, which may reflect differences in local environmental conditions, including potential variation in anthropogenic inputs relative to pumps 1 and 2. Similar location-dependent variation in viral diversity has been reported for groundwater, where viral richness and Shannon index differed among different locations reflecting the differences in the physicochemical properties of the studied groundwater [16].

Beta-diversity analysis showed tight clustering of replicates from each pump separate from those of the other pumps, indicating site-specific virome composition for each pump. This pattern is consistent with groundwater studies reporting site-specific viral communities, including Florida springs where viromes differed among springs despite sharing the same aquifer, likely because of the differences in land use in each location [62], and the multi-well study in the Hainich Critical Zone Exploratory, where virome composition differed based on well location and sampling year [63]. In contrast, very low Bray-Curtis dissimilarities between nearby groundwater sites near a dumpsite suggest that strong hydrological connectivity can homogenize viromes, highlighting that groundwater viral beta diversity depends on local flow and inputs [64].

Host prediction indicated that the identified groundwater viruses primarily target Pseudomonadota (Proteobacteria) as well as Actinomycetota, Bacteroidota and Bacillota (Firmicutes) across all pumps, consistent with the microbial profile detected in the microbial metagenomes [20]. In addition, CPR/Patescibacteriota were predicted as potential viral hosts in pumps 1 and 3. Studies spanning different hydrogeological settings and contamination contexts, including large-scale groundwater datasets [6], long-term contaminated groundwater [65], and the global freshwater virome meta-analysis [66], consistently report that viruses with confident host assignments are dominated by Proteobacteria and frequently accompanied by Bacteroidota/Actinomycetota, with recurrent (often site-dependent) associations to CPR/Patescibacteria in groundwater systems. The predominance of Patescibacteria in certain groundwater environments was previously suggested to be attributable to their ability to thrive well in oligotrophic environments [67].

Correlation and network analyses suggested a close coupling between bacterial communities and their associated viruses in the studied groundwater samples. Across all pumps, CLR-transformed genus-level abundances were strongly positively correlated between the most abundant bacterial genera and viruses potentially infecting them (Pearson *r* = 0.73–0.80; Spearman ρ = 0.65–0.79). In the bipartite network, *Mycobacterium* and *Acidovorax* emerged as core hubs, suggesting that a limited set of key host genera persistently associate with their putative viruses, appearing as stable interaction hubs throughout the three pumps. This pattern supports the possibility of structured virus–host associations in groundwater, although it should be cautiously interpreted because iPHoP-based host assignments are probabilistic, and concordant abundance patterns may also arise from shared environmental influences. These findings align with MAG-resolved groundwater data showing that viral and host abundances are strongly correlated and that viral populations can match or exceed host abundances across many lineages, implying a high potential for phages to regulate groundwater microbial communities [6].

Predicted viral lifestyles differed slightly but significantly among pumps. While pump 2 seemed to have roughly equal proportions of lysogenic and strictly lytic genetic markers, lysogenic markers were relatively more abundant in pump 1, and that ratio was reversed in pump 3. This pattern is ecologically consistent with the microbial data: pumps 1 and 2 harbored less diverse microbial communities dominated by fewer taxa, conditions under which lysogeny is thought to be selected as a viral persistence strategy [68]. In contrast, the higher microbial diversity and evenness in pump 3 likely support more frequent lytic interactions, promoting top-down control and preventing competitive dominance of individual taxa [69], although experimental validation would be required to confirm this interpretation. Of note, these lifestyle assignments are model-based predictions and should be interpreted cautiously. The lack of phylogenomic segregation between strictly lytic and temperate phages further suggests that lifestyle is a flexible trait within groundwater viral communities, potentially influenced by microbial community structure and environmental conditions [70].

AMGs were detected in all three pumps, but their composition and abundance varied from one pump to another. Across pumps, AMGs related to amino acid metabolism were dominant, consistent with the central role of these pathways in supporting viral replication [71, 72]. At the same time, pump-specific AMG repertoires point to localized viral adaptations. Energy metabolism–related AMGs were restricted to pump 2, while pump 3 showed a broader suite of cofactor and vitamin metabolism genes, including folate and pterin biosynthesis. Consistent with this pattern, Gios and colleagues [16] reported that the groundwater AMGs identified in their study predominantly belonged to amino acid and carbohydrate metabolism (especially nucleotide sugar/glycan). Interestingly, the detection of anaerobic dimethyl sulfoxide (DMSO) reductase subunit A (K07306) in pump 2 is notable, particularly since the host of the viral contig harboring this gene was assigned with high confidence to the genus *Thiothrix*. *Thiothrix* is a facultatively anaerobic genus that can be either autotrophic, oxidizing reduced sulfur compounds to sulfate, or heterotrophic. As a facultative anaerobe, it can use oxygen, nitrate, or thiosulfate as terminal electron acceptors [73]. Although we could not find evidence in the literature that *Thiothrix* can use DMSO as a terminal electron acceptor, we hypothesize that the identified AMG (*dmsA*) may enhance the respiratory flexibility of *Thiothrix* by potentially expanding its range of electron acceptors, particularly under oxygen-limited conditions. Viral encoding of respiratory AMGs has been proposed as a viral strategy to enhance host energy metabolism during infection, thereby sustaining viral replication in energy-limited environments [71]. Similar links between viral AMGs and sulfur metabolism have been documented in aquatic and subsurface systems, where viruses carry genes involved in host sulfur redox transformations [4, 6].

Functional annotation of viral contigs was only able to annotate ~ 40% of viral genes, leaving > 60% with unassigned functions. This phenomenon, dubbed as the “viral dark matter,” is a pervasive attribute of virome datasets globally. It highlights the current limitations of reference databases, especially for environmental viruses, and is consistent with the lack of central metabolism or protein synthesis in viruses, which are functional categories that are almost always well annotated in the genomes of cellular organisms. Studies across aquatic, soil, human gut and extreme environments consistently find that a large fraction of viral genes lack homologs with known function (reviewed in [57]). Nevertheless, the annotated genes show a stable and consistent representation of core phage structural and replication modules (head, tail, DNA/RNA metabolism). This stable representation across varying levels of viral diversity confirms that they are core, essential functional genes, maintained independently of diversity shifts triggered by local ecological conditions. Moreover, the relatively higher proportion of genes involved in integration and excision in pumps 1 and 2 is consistent with the enrichment of these two pumps with viral contigs from temperate phages, as these functions are essential for lysogeny and prophage maintenance [74].

Taken together, these results highlight the potential role of groundwater viruses in shaping the microbial community structure and function. Viral community composition, lifestyle strategies, host associations, and functional potential all varied among pumps and were consistent with differences in local microbial communities and environmental context. Because this study did not include direct physicochemical measurements (e.g., nitrate, sulfate, DOC, redox indicators, conductivity, or temperature), links between virome structure and local contamination or hydrochemical status remain suggestive and require future validation. Nevertheless, the observed patterns align with growing evidence that groundwater viromes are not only diverse and novel but also ecologically meaningful, with potential roles in shaping microbial dynamics, nutrient cycling, and biochemical processes across diverse subsurface environments.

## Supplementary Information

Below is the link to the electronic supplementary material.

Supplementary Material 1 (PDF 2.89 MB)

## Data Availability

The raw sequence reads for this study are available through the NCBI Sequence Read Archive (SRA) with BioProject accession PRJNA1196429.
