# Supplementary material for: Metagenomic Analysis of Rural Groundwater Viromes Reveals Bacteriophage Contributions to Groundwater Microbial Ecology
Source: Microb Ecol. 2026 Jun 24;89:132. doi: 10.1007/s00248-026-02818-y (PMC13303688; doi:10.1007/s00248-026-02818-y)
Supplement: Supplementary file 1 [file 248_2026_2818_MOESM1_ESM.pdf]

Metagenomic analysis of rural groundwater viromes reveals bacteriophage contributions to groundwater microbial ecology

Supplementary Information

Manar-Aleslam M. Mattar[1], Walaa A. Eraqi [2], Mohamed Bakr Zaki [3,4], Akram M. Elkashlan [3], Khaled A. M. Abouzid [5], Ramy K. Aziz [2,6], Aymen S. Yassin [2]*, Ali H. A. Elbehery [1]*

[1] Department of Microbiology and Immunology, Faculty of Pharmacy, University of Sadat City, Sadat City, Egypt
[2] Department of Microbiology and Immunology, Faculty of Pharmacy, Cairo University, Cairo, Egypt
[3] Department of Biochemistry, Faculty of Pharmacy, University of Sadat City, Sadat City, Egypt
[4] Department of Biochemistry, Faculty of Pharmacy, Menoufia National University, km Cairo-Alexandria Agricultural Road, Menofia, Egypt
[5] Department of Pharmaceutical Chemistry, Faculty of Pharmacy, Ain Shams University, Cairo, Egypt
[6] Biosciences Research Laboratories, MARC for Medical Services and Scientific Research, 6th of October City, Egypt

***Corresponding authors**:
ali.elbehery@fop.usc.edu.eg
aymen.yassin@pharma.cu.edu.eg

Supplementary Tables

Supplementary Table 1. Viral contig quality statistics

Pump	Com-plete (n)	High-Quality (n)	Medium-Quality (n)	Low-Quality (n)	Mean length, complete (bp)	Length range complete (bp)	Mean length, high (bp)	Length range high (bp)	Mean length, medium (bp)	Length range medium (bp)	Mean length, low (bp)	Length range low (bp)
**Pump 1**	4	26	41	1785	48,545	33,908-65,168	37,278	6,400-66,174	33,013	4,625-171,337	3,364	1,162-134,126
**Pump 2**	3	15	28	1300	63,368	42,625-79,946	40,740	6,168-77,207	35,180	2,286-117,190	3,253	2,283-58174
**Pump 3**	6	6	43	6275	62,579	33,672-166,742	38,659	35,731-41,608	32,348	2,629-83,758	2,690	2,891-49,908

Supplementary Table 2. Viral taxonomy statistics

Pump	Phylum		Class		Order		Family		Genus		Species	
	C	U	C	U	C	U	C	U	C	U	C	U
**Pump1**	1,928	82	1,927	83	71	1,939	266	1,744	602	1,408	707	1,303
**Pump2**	1,472	52	1,475	49	52	1,472	239	1,285	463	1,061	527	997
**Pump3**	4,772	259	4,772	259	291	4,740	914	4,117	1,771	3,260	1,875	3,156

Number of contigs classified at each taxonomic rank. C, Classified; U, Unclassified.

Supplementary Figures

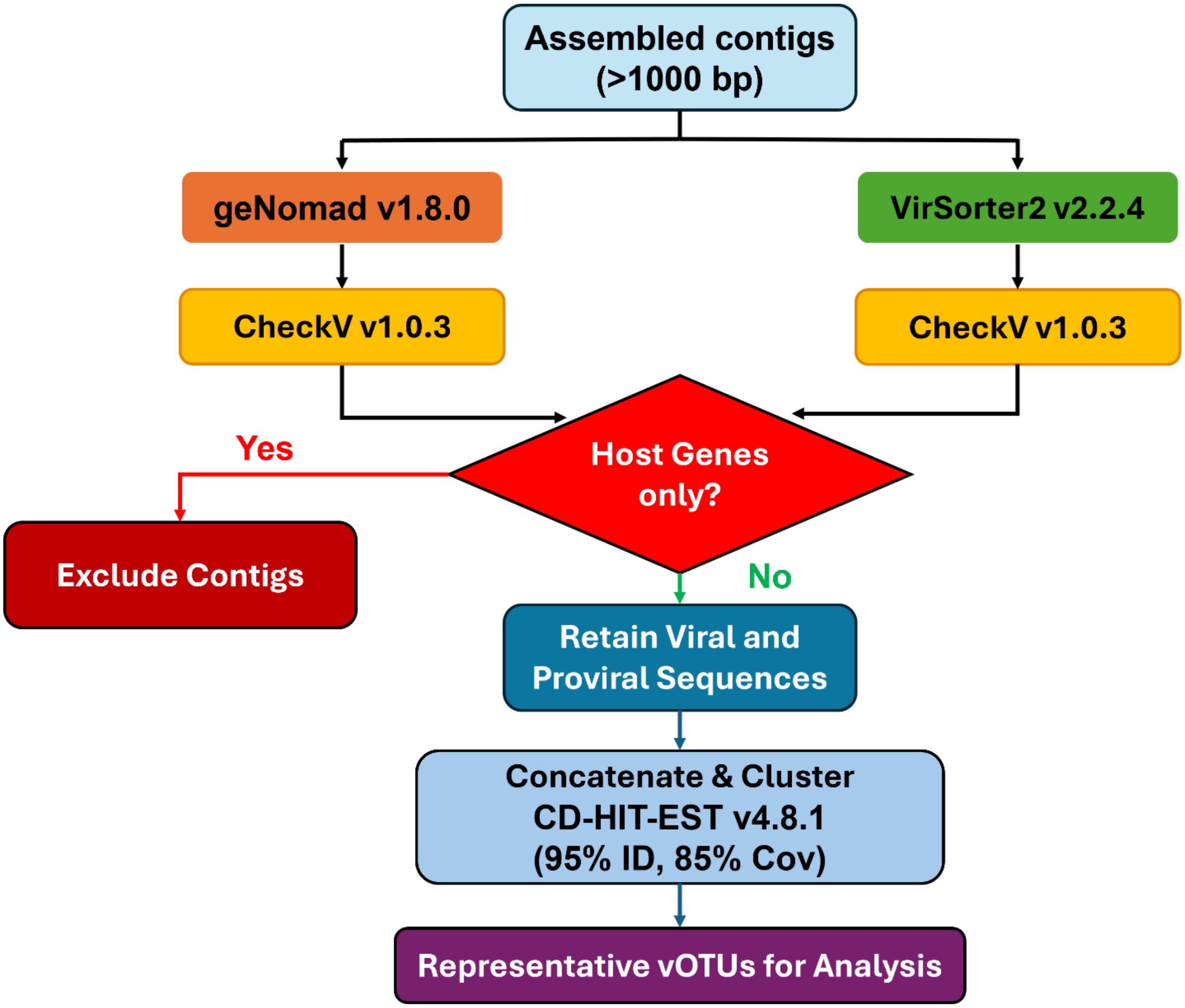

Supplementary Figure S1. Workflow for viral contig identification, filtering, and clustering.

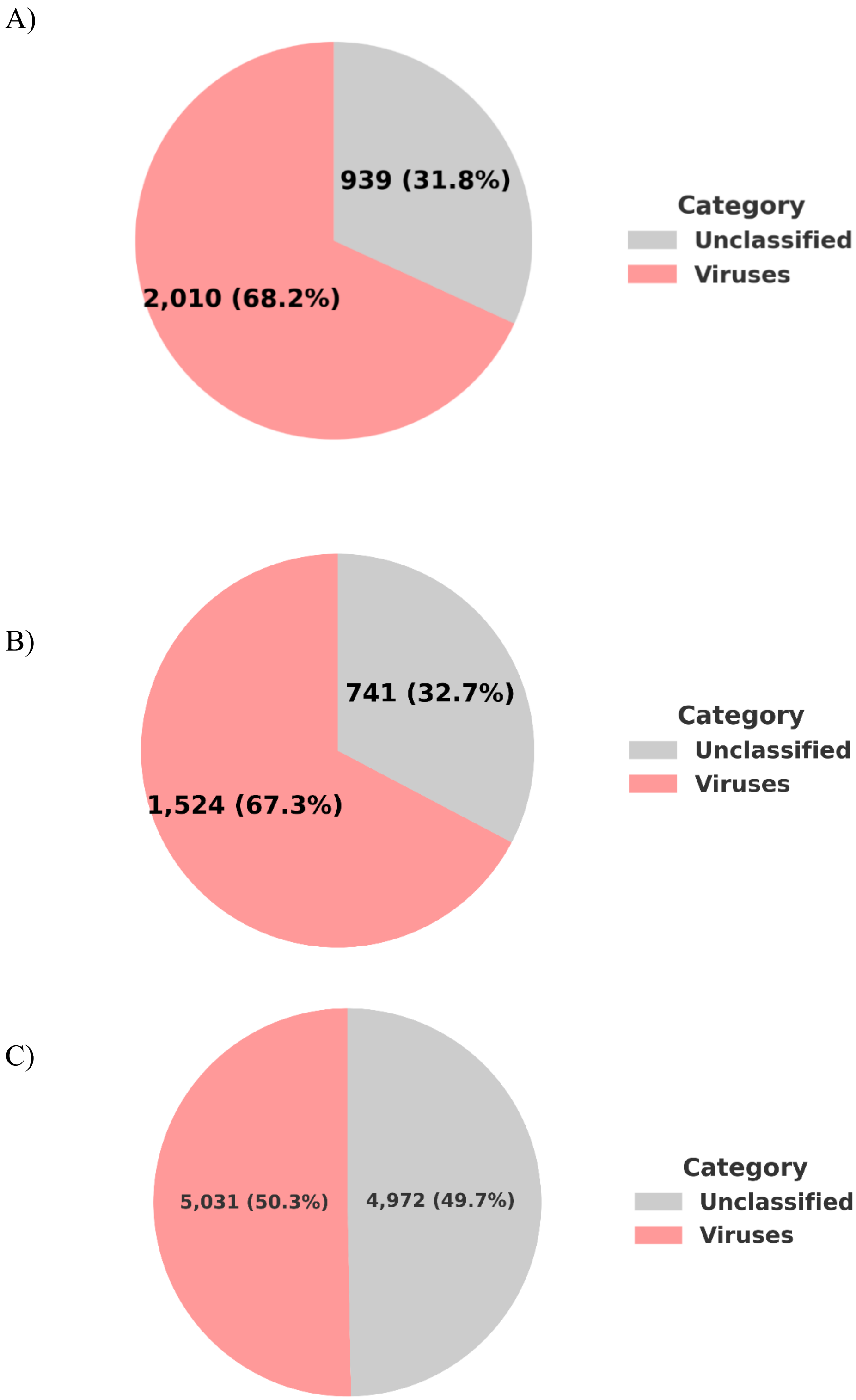

Supplementary Figure S2. Pie chart showing superkingdom-level classification of viral contigs across the three pumps, A) pump 1, B) pump 2, C) pump 3.

A)

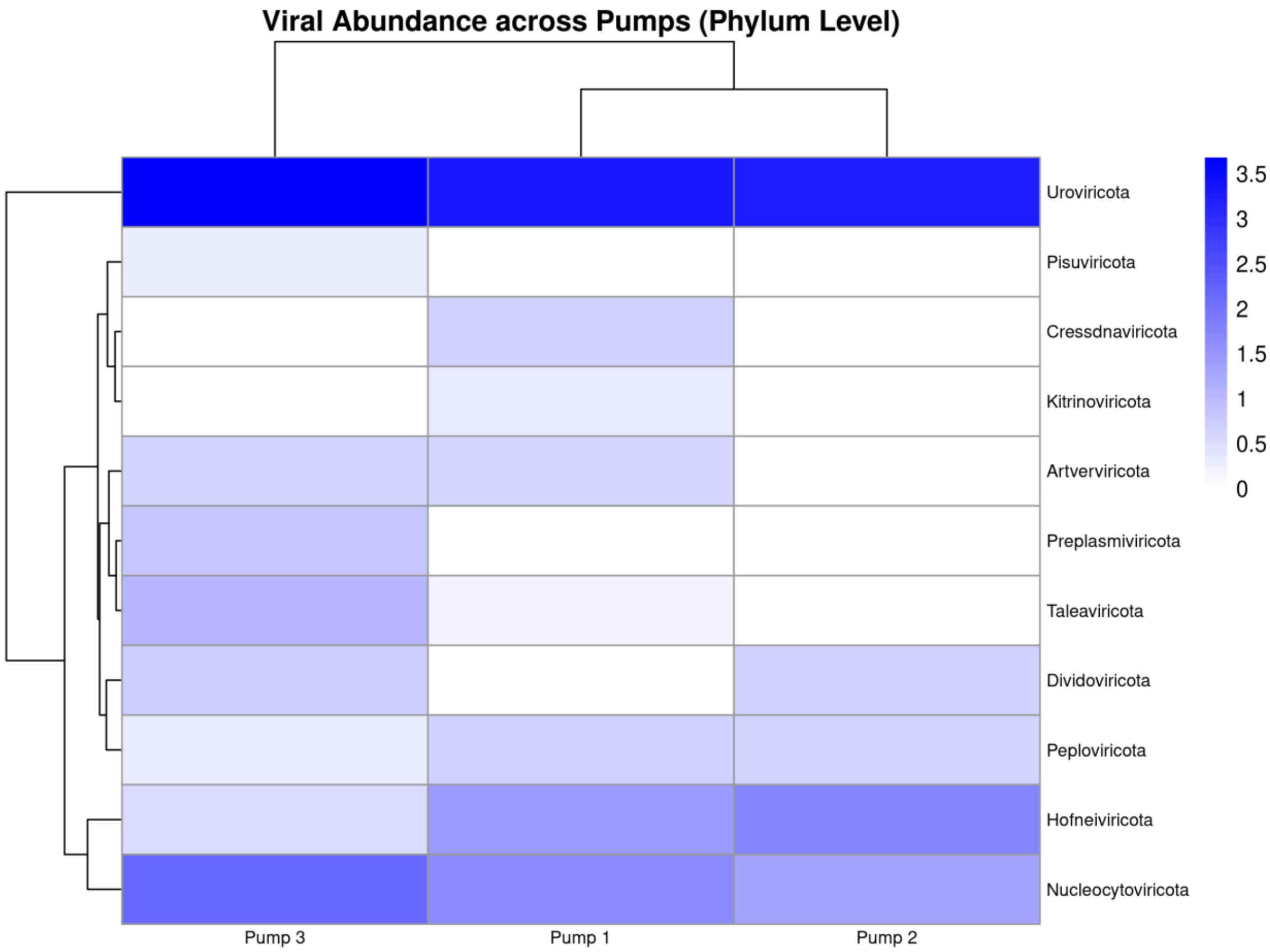

Supplementary Figure S3.A. Heatmap illustrating the relative abundance of taxonomically classified viral contigs in **pump 1, pump 2, and pump 3** at the phylum level

B)

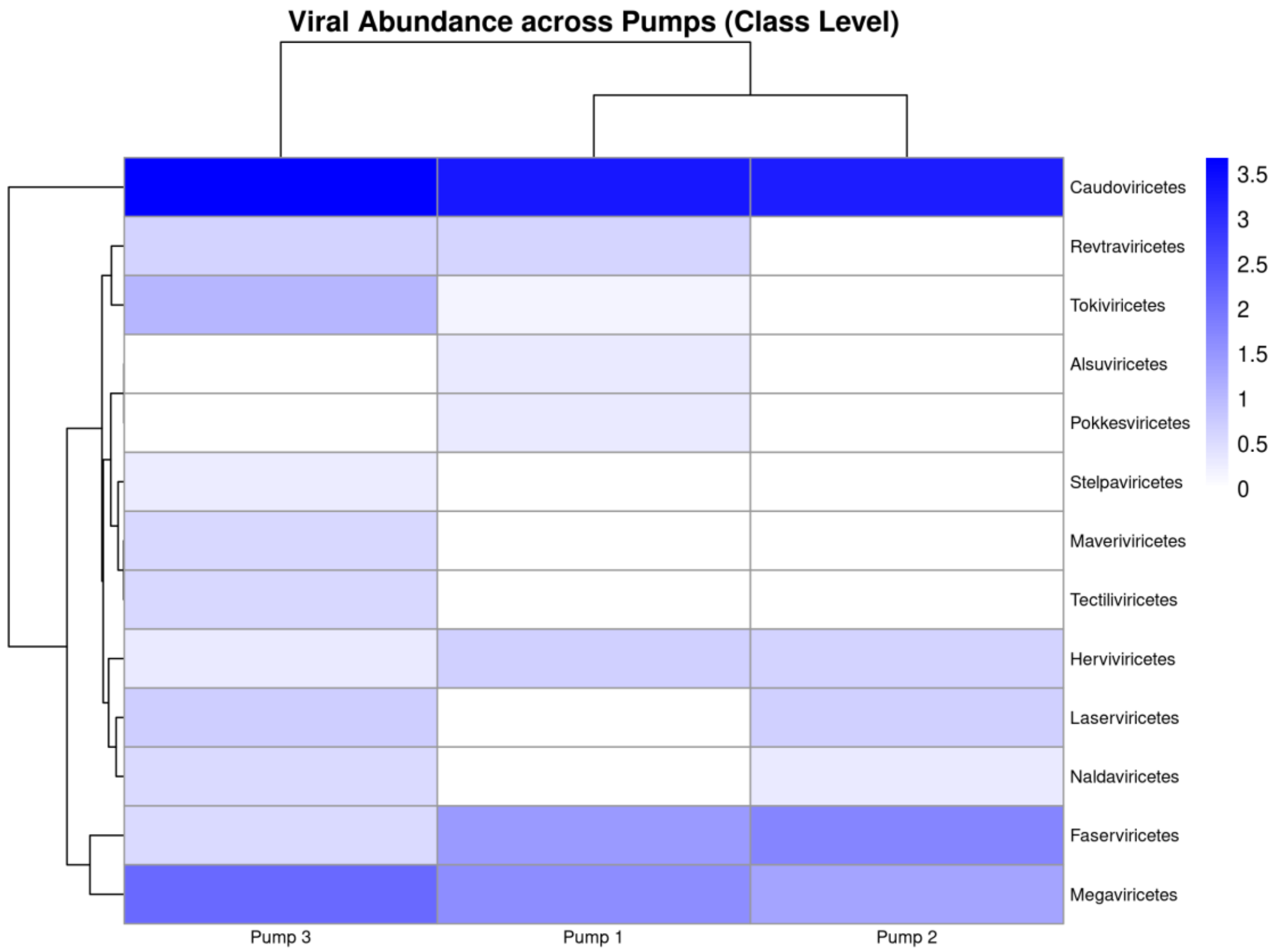

Supplementary Figure S3.B. Heatmap illustrating the relative abundance of taxonomically classified viral contigs in **pump 1, pump 2, and pump 3** at the class level

C)

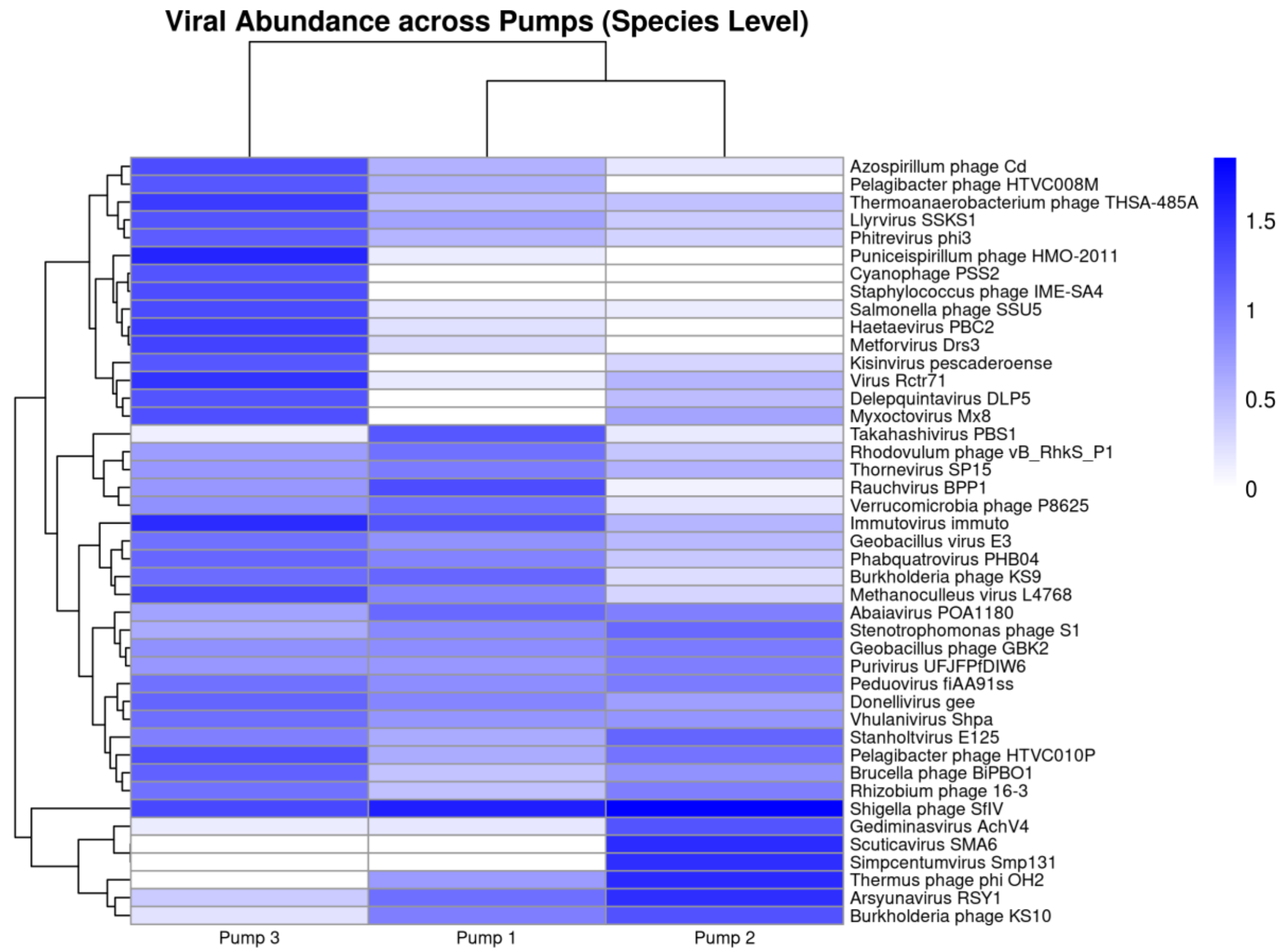

Supplementary Figure S3.C. Heatmap illustrating the relative abundance of taxonomically classified viral contigs in **pump 1, pump 2, and pump 3** at the species level.

A)

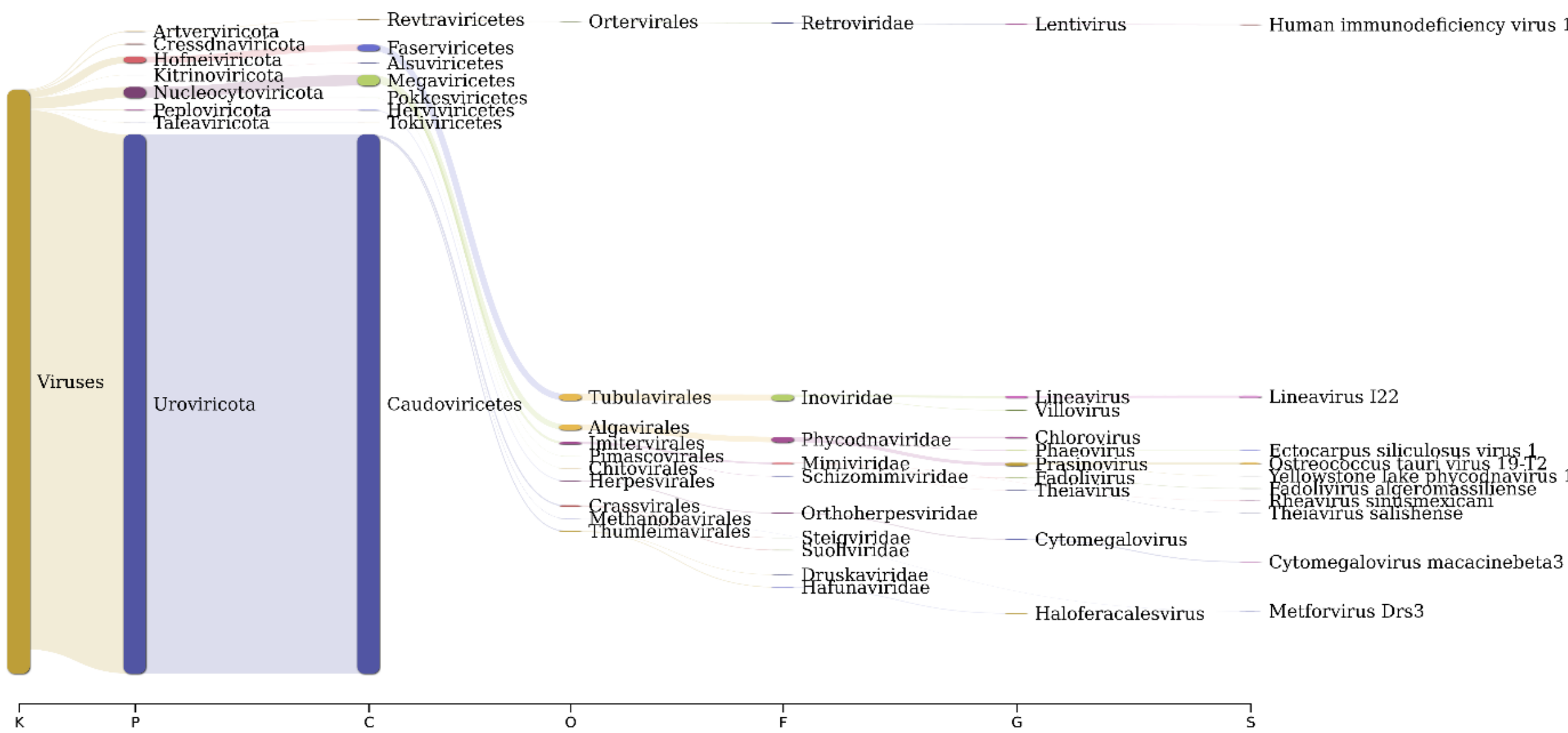

B)

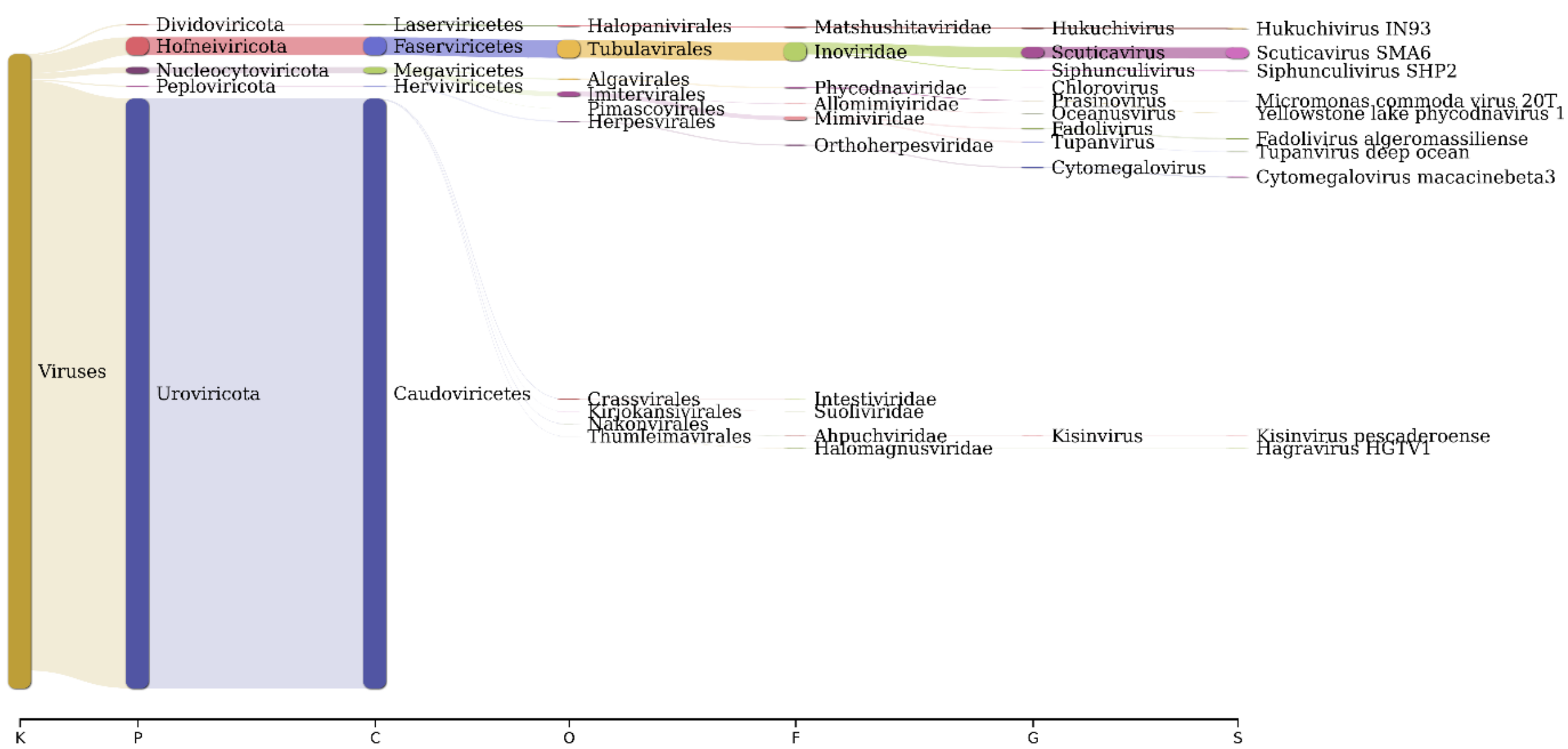

Supplementary Figure S4. Sankey diagrams (generated by Pavian) illustrating the taxonomic classification of viral communities at the levels of phylum, class, order, family, genus, and species for: A) pump 1, B) pump 2, and C) pump 3

C)

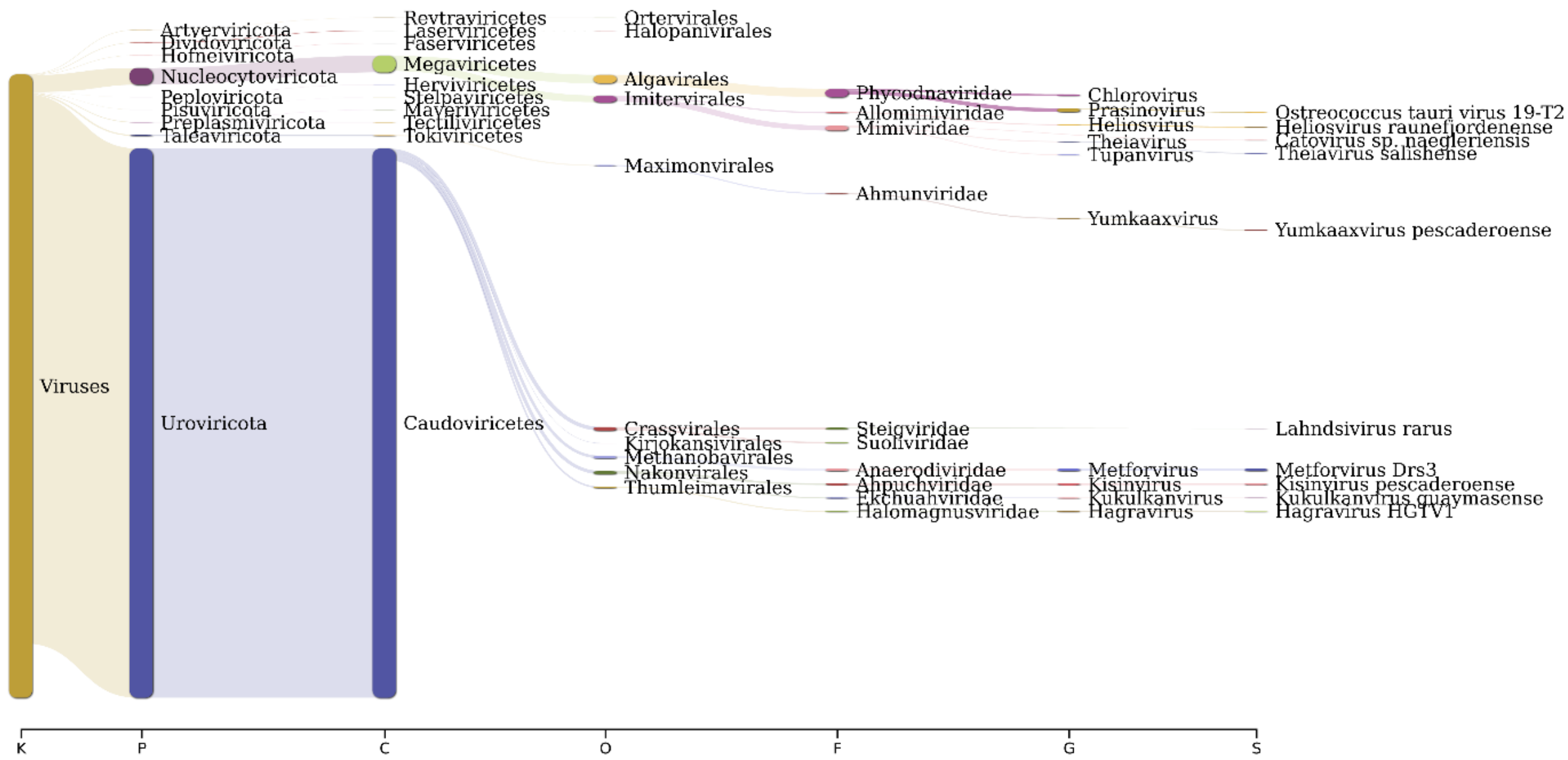

Supplementary Figure S4. Sankey diagrams (generated by Pavian) illustrating the taxonomic classification of viral communities at the levels of phylum, class, order, family, genus, and species for: A) pump 1, B) pump 2, and C) pump 3

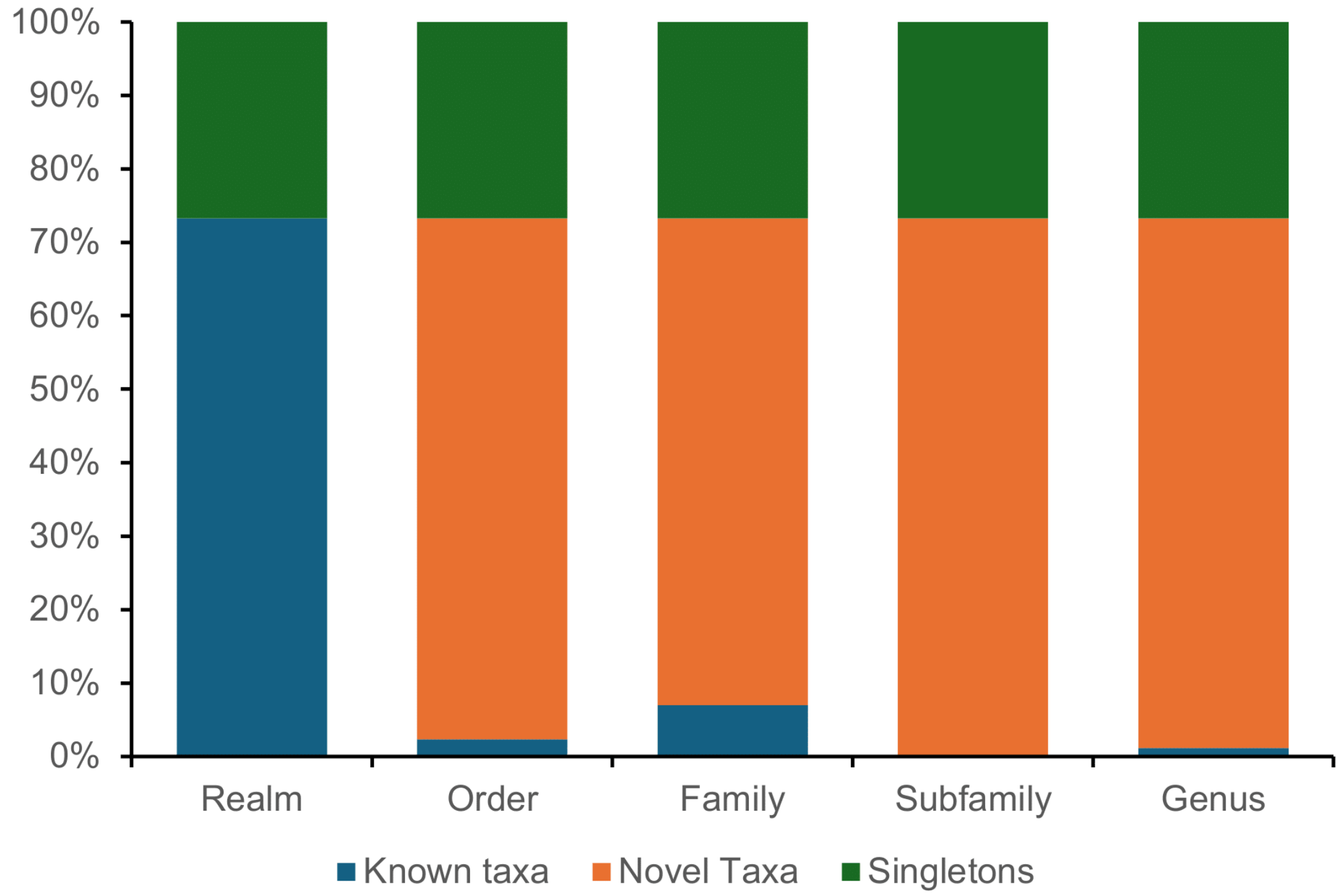

Supplementary Figure S5. Taxonomic novelty among viral genomes inferred using vConTACT3

Supplementary Figure S6. Characterization of the viral dimethyl sulfoxide reductase subunit A (DmsA).

A) Genomic context of the dmsA-containing viral contig.

B) Maximum-likelihood phylogenetic tree of DmsA proteins, with the viral sequence highlighted in red.

C) InterPro-predicted domains and conserved features of the viral DmsA.

A)

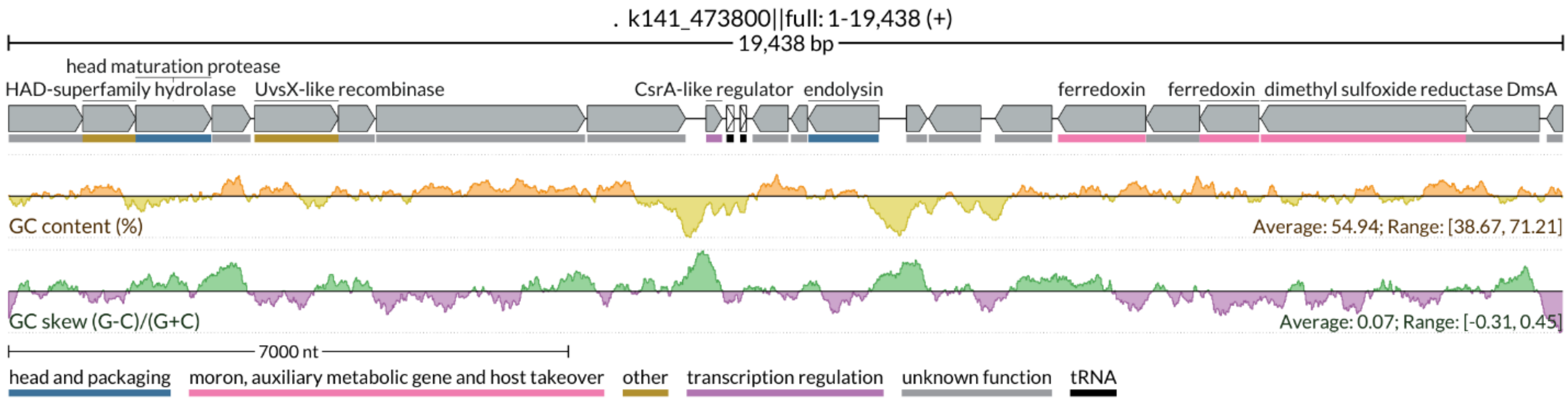

Supplementary Figure S6.A. Genomic context of the dmsA-containing viral contig.

B)

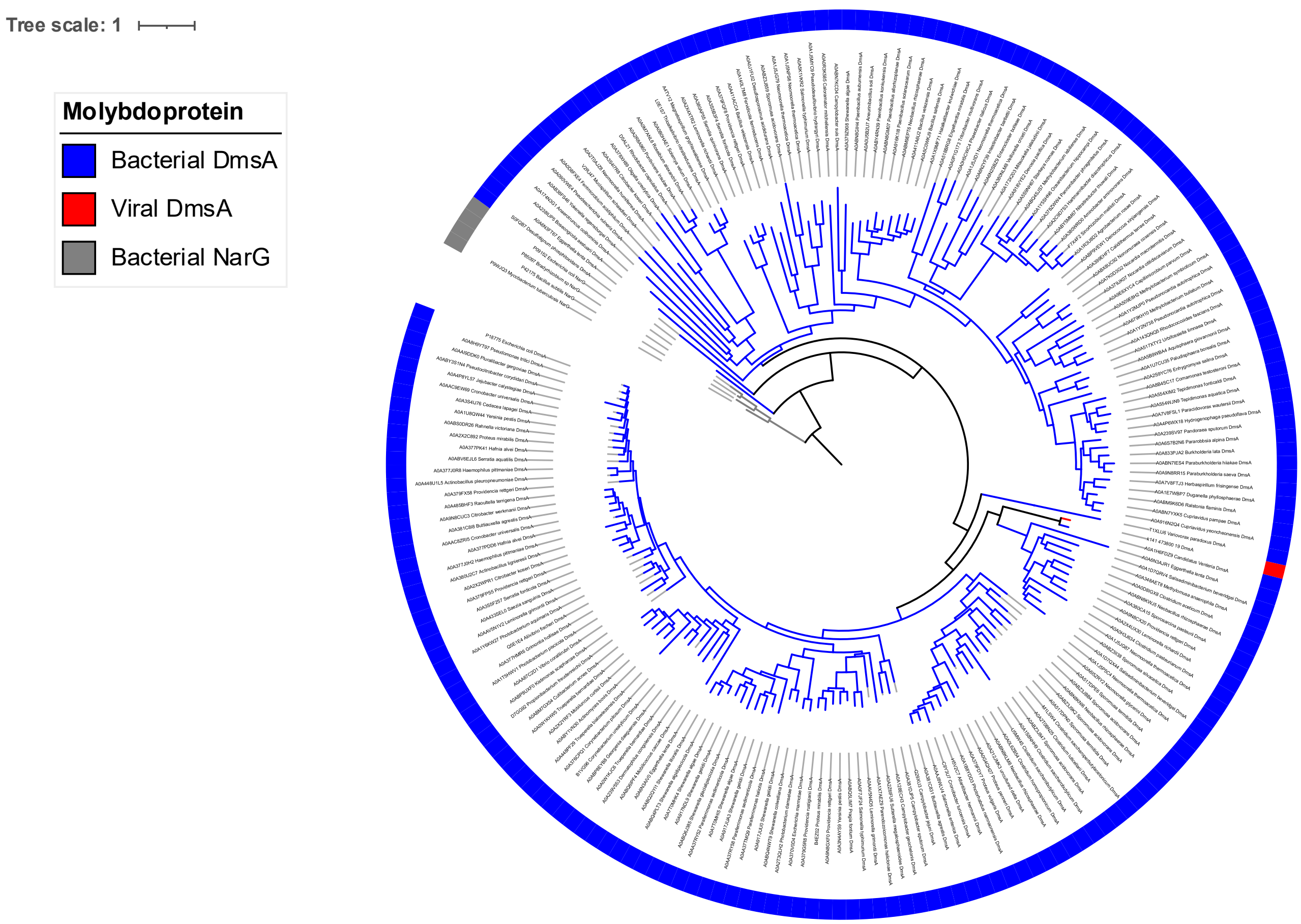

Supplementary Figure S6.B. Maximum-likelihood phylogenetic tree of DmsA proteins, with the viral sequence highlighted in red.

C)

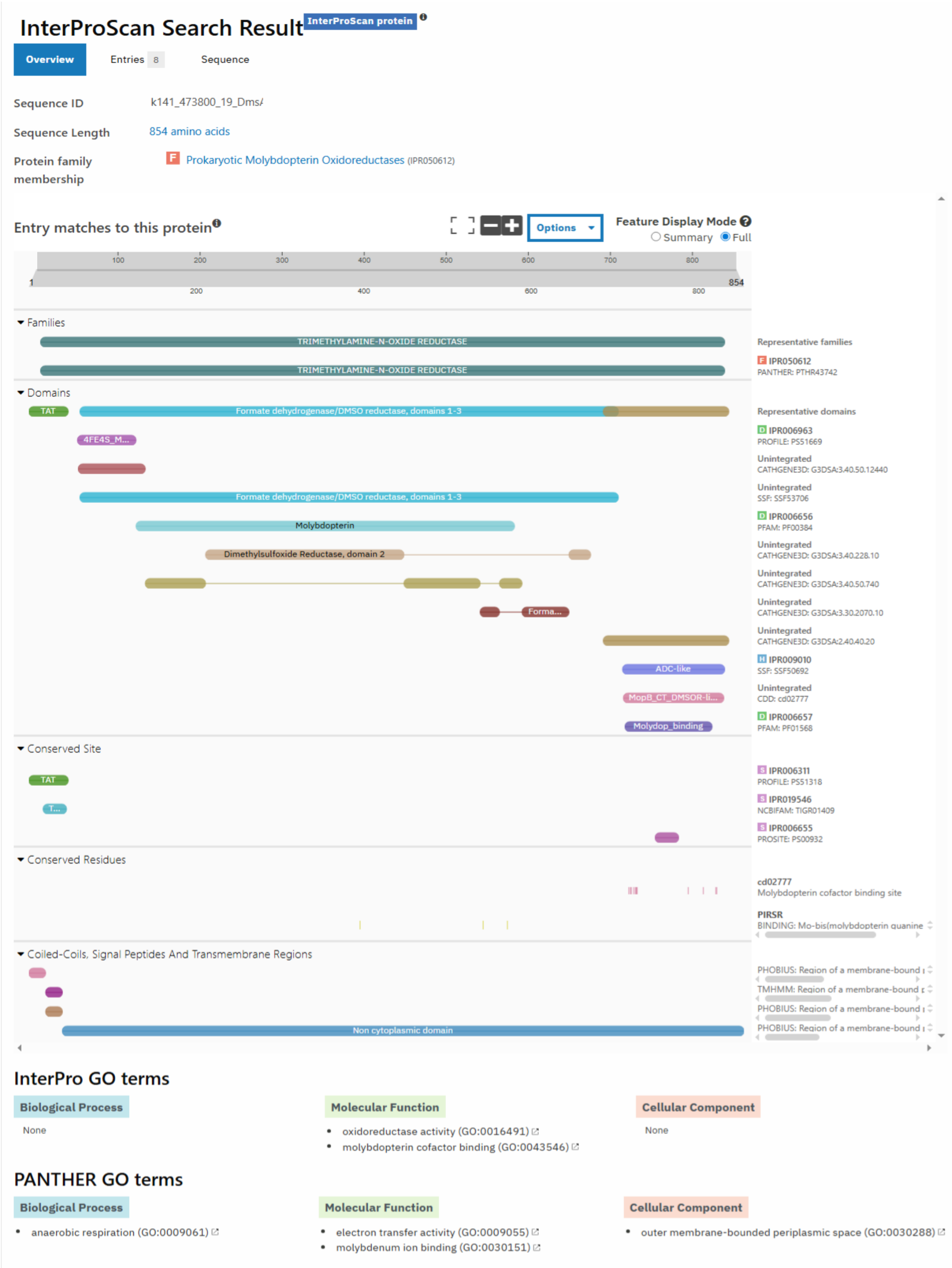

Supplementary Figure S6.C. InterPro-predicted domains and conserved features of the viral DmsA.
